# One trigger, two fates: hemolysate shapes neutrophil extracellular traps (NETs) formation in a context-dependent manner

**DOI:** 10.3389/fimmu.2026.1917185

**Published:** 2026-09-03

**Authors:** Daniel Gucwa, Anna Such, Elzbieta Kolaczkowska

**Affiliations:** 1 Department of Experimental Hematology, Institute of Zoology and Biomedical Research, Faculty of Biology, Jagiellonian University, Krakow, Poland; 2 Doctoral School of Exact and Natural Sciences, Faculty of Biology, Jagiellonian University, Krakow, Poland

**Keywords:** neutrophil, NETs, damage associated molecular patterns, hemolysate, hemoglobin, heme, lipopolysaccharide, inflammation

## Abstract

During systemic inflammation, neutrophil extracellular traps (NETs) capture pathogens but, in the long-term, can cause bystander damage to the vasculature. Another hallmark of sepsis in the blood is erythrocyte damage/alterations, often leading to hemolysis. Here, we report that hemolysate modulates NET formation, depending on the inducing agent. Whereas pharmacological agents, yeast components, and live Gram-positive bacteria either do not alter or somewhat enhance NET release by murine neutrophils in presence of hemolysate, it inhibits lipopolysaccharide (LPS)-induced NET formation. This inhibition depends on the number of erythrocytes undergoing hemolysis, the timing of LPS application, the neutrophil source (healthy/endotoxemic mice), and cell organization (2D/3D conditions). To verify the specificity of NET formation, Peptidyl Arginine Deiminase 4 (PAD4)-deficient mice were employed. To further elucidate the mechanisms of LPS action, separate hemolysate components were studied, namely hemoglobin, heme, and erythrocyte ghosts. Although all three contributed to NET downregulation, hemoglobin was the primary inhibitor of NETs. In line with this, the depletion of hemoglobin from the hemolysate reversed NET release, and in its presence (in hemolysate or pure), less LPS was available to neutrophils. LPS binding by hemoglobin was previously described, but it was reported to rather enhance its biological activity and amplify inflammatory signaling in some contexts. Therefore, we verified whether the process also occurs *in vivo* by intravital microscopy. During endotoxemia accompanied by hemolysis, both neutrophil infiltration and NET formation (relative to the cell number) were reduced. Taken together, our studies show that hemolysis diminishes Gram-negative bacteria-induced neutrophil activation and NET release.

## 1 Introduction

Neutrophils are the first immune cells to respond to any insult to the organism (1). They fight pathogens by releasing their antimicrobial content (2) or by actively taking them up via phagocytosis (3) and they further eliminate them in phagolysosomes via reactive oxygen species (ROS) (4). Moreover, neutrophils form neutrophil extracellular traps (NETs), which limit the spread of an inflammatory outbreak and might, in some scenarios, kill trapped pathogens (1, 5). NET structure consists of decondensed DNA fibers along with attached proteins, mostly of granular (e.g., neutrophil elastase, NE) or nuclear origin (e.g., histones). NETs are formed in inflamed tissues and during systemic inflammation, in the vasculature (6, 7) where they are additionally exposed to other factors/processes occurring in the blood. One of them is hemolysis, which arises in various diseases (8–11), in the course of sepsis (12) or even as a result of medical procedures (13, 14), during which erythrocyte components which belong to danger-associated molecular patterns (DAMPs; such as hemoglobin, heme, and cell membrane residues) are released extracellularly (14).

The impact of free hemoglobin on the immune system remains poorly understood. Although some studies have demonstrated its effects on leukocyte activity (15, 16), hemoglobin-derived heme has been consistently identified as a major proinflammatory molecule (17, 18). Importantly, heme, but not hemoglobin, induces NET formation in isolated human neutrophils via ROS generation (19). It has also been reported that free heme might contribute to susceptibility to infections by inducing heme oxygenase 1 (HO-1) expression (20). Furthermore, heme, through cytoskeletal remodeling, impairs neutrophil phagocytic ability (21, 22). Importantly, similar properties were not observed for hemoglobin or free iron (21). The majority of studies on hemolysate have focused on its individual components (yet not all); however, neutrophils are exposed to its full content in blood. In addition to hemoglobin and heme/iron (21), the hemolysate contains cell membrane remnants (cell ghosts) that carry various surface molecules and cytoskeletal components, all of which may affect cellular responses. Additionally, erythrocytes may shed extracellular vesicles, and our group has shown that they suppress NET formation via the Siglec-E receptor (6). Intact human erythrocytes exhibit similar properties via the analogous Siglec-9 pathway (23, 24).

Therefore, the aim of the current study was to investigate the impact of whole hemolysate on neutrophils and their capacity to form NETs, and to identify the major effector molecules within the hemolysate. Because neutrophils function not only in the vasculature but also migrate to tissues during inflammatory reactions, we compared their viability in both 2D and 3D cell culture models. Herein, we report that whole hemolysate significantly limits LPS-induced NET formation but increases NET formation induced by PMA or calcium ionophore (CI), other common NET stimulants. The LPS-induced effect is due to sequestration of LPS by components of the hemolysate, primarily hemoglobin. This effect was confirmed to operate *in vivo*, as pharmacological induction of hemolysis during endotoxemia limited NET formation.

Altogether, our findings provide new, previously unreported details on the impact of hemolysate on neutrophil immune activity during inflammation and underscore its dual effect, which depends on the immunostimulant, with lipopolysaccharide standing out.

## 2 Materials and methods

### 2.1 Mice

C57BL/6J male mice were purchased from Charles River Laboratories (Sulzfeld, Germany, via AnimaLab, Poland), and peptidyl arginine deiminase 4 (PAD4) knockout (KO) mice were purchased from the Jackson Laboratory (Bar Harbor, ME). PAD4^-/-^ mice were bred in-house. All mice were housed under standard environmental conditions (temperature 21-22°C, illumination on a 12:12 h light/dark cycle) with access to commercial food (Maintenance Diet 1324; Altromin, Germany) and tap water (*ad libitum*). All procedures and methods were approved by the Local Ethical Committee No. II in Krakow (294/2017, 22/2023 and 22A/2023) and were performed in accordance with the Guide for the Care and Use of Laboratory Animals (Directive 2010/63/EU of the European Parliament), Polish legislation (Act 266/2015, updated in 2021), and institutional animal care policies.

### 2.2 Erythrocyte isolation and lysis

Mice were anesthetized with a mixture of ketamine hydrochloride (200 mg/kg b.w.; Biowet Pulawy, Pulawy, Poland) and xylazine hydrochloride (10 mg/kg b.w.; aniMedica, Südfeld, Germany). Whole blood was collected from the heart using a syringe (27G needle, dicoSULIN, Zarys, Poland) and immediately diluted with an equal volume of HBSS(-) (ThermoFisher Scientific, USA). Erythrocytes were isolated by Percoll gradient centrifugation (65% and 72%, respectively) prepared with a glass Pasteur pipette (DWK Life Sciences, Germany). The cell suspension was layered over the gradient and centrifuged (2700 rpm, 22°C, 35 min). The erythrocyte fraction was collected, suspended in HBSS(-), and centrifuged (1500 rpm, 22°C, 6 min). The pellet was resuspended in 1 ml HBSS(+) (ThermoFisher Scientific, USA), and erythrocytes were counted in a Bürker hemocytometer. Erythrocyte purity was ~99% (6). Subsequently, the cells were centrifuged (3600 rpm, 22°C, 15 min) (25), and the pellets were frozen (-20°C, >24 h). Three different hemolysates were prepared to reflect varying degrees of erythrocyte lysis. Based on reported plasma levels of cell-free hemoglobin in patients with sepsis and other diseases, we selected different numbers of erythrocytes for hemolysate preparation to obtain a range of hemoglobin concentrations representing varying degrees of hemolysis. The intermediate condition (2.5×10^6^ containing ~0.3 mg/ml of hemoglobin) was selected to best reflect clinically relevant levels of hemolysis (0.1–1 mg/ml with 0.15-0.4 mg/ml representing the range most frequently reported in patients with sepsis) (26–29), while the lowest and highest conditions were included to extend the experimental range and provide insight into cellular responses under minimal and pronounced hemolytic conditions. Namely, either 0.25×10^6^, 2.5×10^6^, or 25×10^6^ of lysed RBCs were used to obtain 3 different hemolysates. This procedure resulted in temperature-dependent RBC lysis, as confirmed by a colorimetric method that estimated hemoglobin levels (30, 31). Hemolysate from endotoxemic mice was obtained similarly, with mice administered 1 mg/kg body weight (b.w.) of lipopolysaccharide (LPS, *Pseudomonas aeruginosa*, serotype 10.22; Sigma–Aldrich, USA) intraperitoneally in saline, 8 or 24 hours prior to blood collection, to induce systemic inflammation. In experiments conducted in 96-well plates (NEST, China), 3 hemolysate concentrations (obtained from 0.25×10^6^, 2.5×10^6^, or 25×10^6^ erythrocytes) were added to the cells simultaneously with LPS or other stimulants (unless stated otherwise). In some experiments, coverslips (Ø5 mm, Epredia Netherlands B.V., Netherlands) were placed at the bottom of the wells to facilitate immunocytochemical staining and analysis.

### 2.3 RBC ghost preparation

Murine RBC ghosts were prepared by hypotonic lysis following the method of Dodge, Mitchell, and Hanahan, with some modifications (32). Briefly, the erythrocyte fraction was resuspended in PBS, and then 10× volume of ice-cold hypotonic phosphate buffer (5 mM, pH 7.4; 535.19 mg Na_2_HPO_4_ anhydrous + 191.9 mg NaH_2_PO_4_×2H_2_O per liter of distilled water) was added. The mixture was incubated for 30 minutes. Subsequently, the RBC suspension was centrifuged (12000 rpm, 15 min, 4°C). The supernatant was discarded, and the pellet resuspended in the same volume of ice-cold hypotonic phosphate buffer and centrifuged immediately (12000 rpm, 15 min, 4°C). This step was repeated until the pellet was free of hemoglobin. After the final washing step, the supernatant was discarded, and the RBC ghost pellet was frozen at -20°C for at least 24 h (to recreate the conditions of RBC lysis). In some experiments, RBC ghosts were additionally incubated (1 h, 37°C) with neuraminidase (from *Vibrio cholerae*, Sigma-Aldrich, USA) to enzymatically remove surface sialic acids prior to further analysis.

### 2.4 Estimation of hematologic parameters and hemoglobin levels

Hemoglobin levels were assessed using the URIT-5160Vet hematology analyzer (URIT Medical Electronic Co. Ltd, China) and the Drabkin method. All hemolysate samples were diluted to 100 µl before measurement. This method was also used to analyze whole blood collected from healthy mice or from mice treated with phenylhydrazine (PHZ, Thermo Scientific, Germany) (see below). The hematology analyzer measures white blood cell (WBC), red blood cell (RBC), and platelet (PLT) counts by impedance (Coulter principle); hemoglobin concentration is determined calorimetrically with cyanide-free reagents; and a 5-diff WBC differential is obtained by multiangle laser flow cytometry. For the Drabkin method, 10 µl of each hemolysate was added to 2.5 ml of Drabkin reagent (Stamar, Poland). After 5 min of incubation at RT, spectrophotometric measurements (Bio-Tek El800, USA) were performed at 540 nm. Hemoglobin from Biochemtest (Poland) was prepared in the same manner and served as a standard. The concentration of hemoglobin in samples was calculated using the following formula:


$$[HL]=\frac{{OD}_{1}}{{OD}_{2}}\times [HB]g/dl$$

33

Where OD_1_ is the absorbance of each sample, OD_2_ is the absorbance of the standard hemoglobin solution, [HB] is the concentration of the standard hemoglobin solution (165 mg/ml), and [HL] is the concentration of the hemolysate sample. Additionally, absorbance scans (350–650 nm) of hemolysate and hemoglobin were performed at time 0 and after 6 h of incubation.

### 2.5 Isolation of neutrophils

Mice were anesthetized with a mixture of ketamine hydrochloride (200 mg/kg b.w.) and xylazine hydrochloride (10 mg/kg b.w.). Cervical dislocation was then performed, and the femur, along with the tibiae, was isolated. Epiphyses were removed, and bone marrow was flushed into a Petri dish containing ice-cold HBSS(-) using a 25G needle (Terumo Agani, China). Bone marrow was then dissociated using a 20G needle (Terumo Agani, China), yielding a homogeneous suspension. The suspension was centrifuged (1300 rpm, 4°C, 6 min). The pellet was resuspended in 1 ml of 0,2% NaCl (Braun, Germany) and then finely pipetted for 25 s to induce erythrocyte hypotonic lysis. Immediately after, 4 ml of 0.2% NaCl and 5 ml of 1.6% NaCl were added, respectively. Cells were then centrifuged (1400 rpm, 4°C, 7 min) and resuspended in 2 ml of 52% Percoll (GE Healthcare, Chicago, IL, USA). Subsequently, 2 ml of 69% and 78% Percoll, respectively, was added gently with a glass Pasteur pipette, and cells were centrifuged for 30 min (2600 rpm, 4°C). The neutrophil fraction localized between the 69% and 78% Percoll layers was collected with a glass Pasteur pipette. Neutrophils were suspended in HBSS(-) and centrifuged (1500 rpm, 4°C, 6 min). The purity of isolated neutrophils was ~ 95%, as estimated by their morphology with Türk solution (0.01% crystal violet in 3% ethanoic acid; Sigma–Aldrich, USA), and viability was ~ 96% with Trypan blue (Sigma-Aldrich, Saint Louis, MO, USA) (both with Bürker hemocytometer counting chamber). This method was previously confirmed using an anti-mouse Ly6G antibody (BioLegend, San Diego, CA, USA) by flow cytometry (34).

### 2.6 Neutrophil stimulation

Neutrophils (5×10^4^) were seeded and allowed to adhere for 30 min at 37°C in 5% CO_2_ in the dark (Thermo Electron, USA). To induce the inflammatory response, cells were stimulated with LPS at a final concentration of 50 µg/ml and incubated for 6 h (37°C in 5% CO_2_ in darkness). The 6-hour time point was selected because, at this time, NETs are still being produced strongly, and their disintegration has not yet begun. In cell viability studies (PrestoBlue), an additional LPS concentration of 35 µg/ml was used to exclude a possible cell death due to NETosis [lytic death after the NET release (35)]. A range of LPS concentrations was studied to assess their effectiveness in NET release; 35 µg/ml (weaker) and 50 µg/ml (stronger) induced NET formation, whereas higher concentrations (100 and 200 µg/ml) were toxic (**Supplementary Figure 1**). The concentration of 100 µg/ml resulted in not NET-specific extracellular DNA (extDNA) release (necrosis/necroptosis) but the highest does seemed to completely arrest even the DNA release. In flow cytometric cell viability studies 50 µg/ml of LPS was used. The LPS dose was adjusted to mouse sensitivity (versus human), corresponding to doses previously reported to induce NETs in murine neutrophils (36, 37). Some neutrophils were additionally incubated with hemolysates (HL; obtained from 0.25×10^6^, 2.5×10^6^, or 25×10^6^ RBCs) or their components: mouse hemoglobin (HB; 0.03, 0.3 or 3 mg/ml; Cusabio, USA), hemin (10 or 20 μM; Merck, USA), or RBC ghosts (originating from 0.25×10^6^, 2.5×10^6^, or 25×10^6^ RBCs; obtained as described above). In some experiments referred to as “rescue experiments”, higher concentrations of LPS were used: 100 and 200 µg/ml, to co-incubate neutrophils with HL and HB. Additionally, neutrophils were stimulated with phorbol 12-myristate 13-acetate (100 nM, PMA; Merck, USA), N-Formyl-L-methionyl-L-leucyl-L-phenylalanine (1 µM, fMLP; Merck, USA), Zymosan (100 µg/ml; Sigma-Aldrich, USA), A23187 Calcium Ionophore (10 µM; CI; Merck, USA), or MRSA (10:1 bacteria to cell), either alone or in combination with hemolysate or its components. Cells stimulated with hemolysate or its components alone, or not stimulated at all, were used as controls (always indicated in the figure legends). In some cases, as indicated in the figures, the medium was switched from HBSS(+) to RPMI 1640 (with L-glutamine and without phenol red; Thermo Fisher Scientific, USA). RPMI medium was selected for subsequent experiments because preliminary optimization showed that it supported greater NET release than HBSS. To assess LPS binding to neutrophils in the presence of HL, LPS from *Escherichia coli* Serotype: 055:B5 conjugated with Alexa Fluor 568 (Thermo Fisher Scientific, USA) was used. Previously, we showed that both LPS serotypes (*E.coli* 0111:B4 and *P. aeruginosa* serotype 10.22) induce NETs and affect other neutrophil parameters in the same manner (36).

### 2.7 MRSA culture

Methicillin-resistant Staphylococcus aureus (MRSA; TW20, Strain O582; National Collection of Type Cultures, Salisbury, UK) was grown in Brain Heart Infusion (BHI) medium (Sigma-Aldrich, USA). The culture was maintained on an orbital shaker (Ohaus, Switzerland) at 37°C overnight. Subsequently, bacteria were subcultured into fresh BHI under similar conditions for 2 h 20 min to obtain *S. aureus* in mid-log phase growth prior to cell stimulation. The number of CFU was estimated by spectrophotometry, and bacteria were centrifuged (12,000 rpm, 7 min, 21°C) and washed with PBS. Subsequently, bacteria were centrifuged and suspended in HBSS(+).

### 2.8 PrestoBlue viability assay

To assess neutrophil viability by measuring metabolic activity, PrestoBlue reagent (1:10; Thermo Fisher Scientific, USA) was added to each well at the end of each incubation, and the plates were incubated for an additional 30 min at 37°C in 5% CO_2_ in the dark. Subsequently, fluorescence was measured at 535 nm (excitation) and 595 nm (emission) using an Infinite F200 Pro microplate reader (Tecan, Switzerland).

### 2.9 Flow cytometric evaluation of cell viability

To determine cell viability and mode of death, cells were stained with Annexin V-FITC and propidium iodide (PI) using the FITC Annexin V Apoptosis Detection Kit (BD Biosciences) according to the manufacturer’s instructions. Stained cells were analyzed using a Navios flow cytometer (Beckman Coulter, USA). Two-parameter fluorescence dot plots were generated by analyzing 1.5×10^4^ cells. The percentages of positive and negative cells were determined using Kaluza Analysis 2.3 software. As the highest concentration of hemolysate (obtained from 25×10^6^ RBCs) contained membrane ghosts an additional flow cytometric gating strategy was implemented to exclude these particles from the analysis. Importantly, the excluded ghost population did not overlap with the gates defining positively stained neutrophils, confirming that membrane ghosts did not interfere with the quantification of fluorescence-positive cells. In these analyses, neutrophils were stimulated with 50 µg/ml LPS.

### 2.10 Neutrophil phagocytic ability assessment

Coverslips were placed at the bottom of the wells of the 96-well plate. Before cell seeding, latex beads (∅1 µm; 1.25×10^6^ per well; Sigma-Aldrich, USA) were suspended in HBSS(+) and added to the bottom of each well, where they were allowed to settle for 15 min. Neutrophils were incubated with LPS and/or hemolysate as described previously, except that the incubation time was shortened to 2 h. After that, the supernatant was replaced with 100 µl of CellTracker solution (ThermoFisher, USA), and cells were incubated for 30 min (37°C, 5% CO_2_, in darkness) to stain the cytoplasm. Next, cells were fixed with PFA as described above, and the wells were rinsed with 200 µl of PBS, followed by incubation with Hoechst (10 µl; Thermo Scientific, Germany) for 5 min at RT to stain nuclei. Coverslips were then placed on a drop of VECTASHIELD Mounting Medium and prepared for analysis. Images were obtained using a ZEISS EC Plan-NEOFLUAR 20×/0.5 air objective. Neutrophils that phagocytosed beads (CellTracker^+^beads^+^) and the efficiency of this process (number of beads per cell; the phagocytosis index) were estimated with ImageJ software in ≥4 different fields of views (FOVs). To verify the accuracy of the estimation (intracellular presence of beads), 3D reconstructions of neutrophils were performed. Briefly, a series of optical cross sections (z-stacks; 60-80, penetrating up to 100 µm with 1 µm z-step) were acquired using a confocal microscope (whose parameters have been characterized previously) and then overlaid using Imaris v8.4.2 software (Bitplane, Oxford Instruments, UK).

### 2.11 3D culturing

For 3D culture, PuraMatrix reagent was diluted in a sucrose solution to a final concentration of 0.25% PuraMatrix. To establish the physiological pH of the scaffold, 120 µl of HBSS(+) was carefully added, and the scaffold was incubated overnight. The solution was gently removed, leaving only the scaffold in the wells, and neutrophils were immediately seeded onto the scaffold at 5×10^4^ cells per well. No encapsulation was performed, and the cells were allowed to migrate into the scaffold. Neutrophils were stimulated with LPS and/or hemolysate as described above.

### 2.12 Neutrophil extracellular traps and p38 activity evaluation - immunocytochemical staining

Coverslips were placed at the bottom of the wells of the 96-well plate to allow neutrophil adhesion. After incubation with hemolysates, neutrophils were fixed in increasing concentrations of paraformaldehyde (PFA; Thermo Scientific, Germany) in PBS (1, 2, and 3%) for 2, 10, and 20 minutes, respectively, and then washed with 200 µl of PBS (2x). Next, 3% BSA in PBS was added, and the mixture was incubated for 45 min at RT in a humid chamber. Subsequently, citrullinated histone H3 was stained with rabbit polyclonal anti-histone H3 (citrulline R2 + R8 + R17; Abcam, Cambridge, UK) or phosphorylated p38 MAPK with rabbit polyclonal phospho-p38 MAPK (Thr180/Tyr182; Proteintech, USA) antibodies at a 1:200 or 1:150 dilution in 1% BSA, respectively. Additionally, for intracellular staining (phosphorylated p38 MAPK), cells were incubated with 0.1% Triton X-100 for 5 min prior to BSA blocking to facilitate membrane permeabilization. After 18-hour incubation at 4°C in a humid chamber, coverslips were washed twice in PBS and incubated with a secondary Cy3-conjugated goat anti-rabbit IgG antibody (H+L; Jackson Immunoresearch Laboratories, Ely, UK) diluted 1:300 in 1% BSA. After 1 h of incubation at RT in the dark, cells were washed once with PBS. Finally, fluorescent DNA dye Sytox green (5 µM; Invitrogen, Carlsbad, CA, USA) was added and incubated for 5 min in the dark to visualize extDNA. After a final wash in PBS, coverslips were placed in a drop of VECTASHIELD Mounting Medium (Vector Laboratories, Burlingame, CA, USA) and analyzed with a confocal microscope. Images were acquired using a ZEISS EC Plan-NEOFLUAR 20×/0.5 air objective and analyzed with ImageJ (NIH, USA). The presence of extDNA and citrulinated histone H3 (citH3) was quantified and expressed as a percentage of the covered area across ≥ 4 FOV.

### 2.13 Time-dependent impact of hemolysate incubation on NET formation of LPS-primed neutrophils

To determine the timing of hemolysate’s impact on neutrophils, hemolysate and LPS were added in different orders and at different time points. In particular, (i) hemolysate and LPS were added simultaneously; (ii) hemolysate was added to neutrophils 0.5, 2, or 4 h after LPS; or (iii) LPS was added 0.5, 2, or 4 h after hemolysate.

### 2.14 Scanning electron microscopy

To visualize NET formation in greater detail, Scanning Electron Microscopy (SEM) was performed. Neutrophils (3×10^5^ cells/well) were seeded onto uncoated coverslips (**∅**13 mm, Epredia Netherlands B.V., Netherlands) in 24-well plates and allowed to adhere for 30 min (37°C, 5% CO_2_). Cells were incubated with LPS and/or HL (from 2.5×10^6^ RBCs) for 6 h, then fixed in 4% paraformaldehyde in PBS for 15 min. Next, cells were washed with PBS (three times) and dehydrated with ethanol at increasing concentrations (15–100%). They were then immersed for 30 min in a 1:1 mixture of 100% ethanol/acetone. Subsequently, specimens were placed in 100% acetone overnight, then dried in an Anderson critical-point dryer (CPD E3000, Quorum Technologies, UK) using CO_2_. Finally, specimens were coated with a 10 nm layer of gold (JFC-1100E, Fine Coat, JEOL, Japan) and placed in the Scanning Electron Microscope chamber (JSM5410, JEOL, Tokyo, Japan). Images were acquired in secondary-electron mode with an accelerating voltage of 15 keV, a Spot Size of 70 nm, and a Working Distance of 15 mm.

### 2.15 Blockage of HO-1

After cell adhesion (37°C, 5% CO_2_, in darkness, for 30 min), tin protoporphyrin IX dichloride (SnPP, Santa Cruz Biotechnology, Dallas, TX, USA) was added to block HO-1 catalytic activity (final concentration of 20 µM) (38). Incubation then continued for 1 h, followed by hemolysate and/or LPS stimulation for 6 h.

### 2.16 Blockage of Siglec-E signaling

Siglec-E blocking antibodies (3.8 µg/ml; Ultra-LEAF™ purified anti-mouse Siglec-E antibody, M1305A02, BioLegend, San Diego, CA, USA) were added to block the Siglec signaling pathway (6). Blocking antibody was added 30 min prior to hemolysate/ghosts RBC and/or LPS stimulation.

### 2.17 Removal of hemoglobin

Hemoglobin was removed from hemolysates using HemogloBind (Biotech Support Group, USA). Briefly, the HemogloBind resin was washed 3 times with HBSS(+) (9000 rpm, 3 min, RT) and then mixed with hemolysate (from the lysis of 2.5×10^6^ RBCs) at a 1:6 ratio. The mixture was centrifuged (9000 rpm, 3 min, RT), and the hemoglobin-free fraction was collected. Alternatively, hemoglobin was removed from the hemolysate with zinc chloride. Briefly, 4 mM ZnCl_2_ was added to the HL, and the mixture was incubated for 5 min. After that, the HL was centrifuged (1500 rpm, 15 min, RT), and the pellet was discarded. Subsequently, the supernatant pH was adjusted to 7.2-7.4 with NaOH and added to neutrophils with or without LPS.

### 2.18 LPS measurement: the rFC assay

To measure LPS levels, the rFC assay (Acro Biosystems, China) was used with minor modifications. Briefly, hemolysate, hemoglobin and BSA were incubated with LPS (50 µg/ml) for 1 h at 37°C in 5% CO_2_ in the dark. Subsequently, HemogloBind was added to each sample, and the mixture was centrifuged (12000 rpm, 3 min, RT). The supernatant was collected and diluted 50,000-fold in endotoxin-free water (provided by the manufacturer), yielding a final LPS concentration of 1 ng/ml. Prior to measurement, recombinant factor C and fluorogenic substrate were mixed 1:1 and added to the samples. Fluorescence was measured at excitation/emission wavelengths of 380/440 nm using a microplate reader at time 0 and after 1 h of incubation (37°C, in the dark). Results were expressed as a percentage of the LPS level in the sample relative to the positive control level.

### 2.19 Hemolytic and endotoxemic model

To induce RBC lysis *in vivo*, mice were injected with PHZ twice: the first dose of 40 mg/kg b.w. was administered at time 0, and 12 h later, a second dose of 60 mg/kg b.w. was administered for 24 h. In some cases, in 36 h blood was collected and centrifuged (4500 rpm, 15 min 4°C) and plasma collected for HB assessment. We verified that level of free HB was around 1.8–2 mg/ml. After a total of 36 h, mice were injected intravenously with LPS (1 mg/kg b.w.) in saline to induce endotoxemia. After 6 h, mice were subjected to intravital imaging. Control mice were injected with vehicle at the same volumes and at the same time intervals.

### 2.20 Intravital microscopy

This technique was performed as described previously (39). Briefly, mice were anesthetized with a mixture of ketamine hydrochloride (200 mg/kg b.w.) and xylazine hydrochloride (10 mg/kg b.w.). Subsequently, the right jugular vein was cannulated to administer anesthetics/antibodies. NETs were stained with anti–histone H2A.X (0.8 µg per mouse, Santa Cruz Biotechnology, USA) conjugated with AlexaFluor 647 using a protein labeling kit (Thermo Fisher Scientific, USA). Kupffer cells (KCs) were stained with anti-F4/80 monoclonal antibody (1.2 μg/mouse, AlexaFluor 488, eBioscience; Thermo Fisher Scientific, USA), and neutrophils were stained with anti-Ly6G monoclonal antibody (1.6 ug/mouse BV421, BioLegend, San Diego, CA). To expose the liver, an incision was made along the abdominal wall. The left lobe of the liver was placed on a saline (0.9%) moistened Kimwipe tissue. A cover slip was placed on the liver lobe, and microscopic observation was performed.

### 2.21 Spinning disk confocal microscopy

NET and phagocytic abilities of neutrophils were imaged with a ZEISS Axio Examiner.Z1 upright microscope equipped with a confocal spinning-disk device DSD2 (with ZEISS EC Plan-NEOFLUAR 10 ×/0.3, ZEISS EC Plan-NEOFLUAR 20 ×/0.5, ZEISS LD Plan-NEOFLUAR 40 ×/0.6, and 63 ×/0.75 air objectives) and a metal halide light source (AMH-200-F6S; Andor, Oxford Instruments, Abingdon, UK). Fluorescence was detected with a 5.5-megapixel sCMOS camera (Zyla 5.5, Andor, Oxford Instruments, Abingdon, UK). For excitation, the following filters were used: DAPI: 390/40 nm; GFP: 482/18 nm; RFP: 561/14 nm. For visualization, the respective emission filters were: DAPI: 452/45 nm; GFP: 525/45 nm; RFP: 609/54 nm. The microscope was operated using iQ 3.6.1 acquisition software (Andor, Oxford Instruments, Abingdon, UK). Imaris v8.4.2 (Bitplane, Oxford Instruments, UK) was used to assess phagocytosis of latex beads or LPS. This software creates a three-dimensional image by superimposing multiple cross-sectional images (so-called Z-stacks) taken under a fluorescence microscope equipped with a confocal module. For this purpose, a series of 60–80 cross-sectional images was taken through the microscopic specimen, imaging up to 100 µm of the specimen’s thickness along the z-axis. Fluorescence images were taken separately for each channel (DAPI, GFP, RFP, and Cy5) and then merged into a single 3D reconstruction.

### 2.22 Statistics

All data are presented as mean values ± SD. The normality of the data was assessed using the Shapiro–Wilk test. To evaluate statistical significance, one-way ANOVA with Bonferroni multiple-comparison *post hoc* test was performed. For data that did not meet the normality assumption, the nonparametric Kruskal–Wallis test followed by Dunn’s multiple-comparison *post hoc* test was used. Statistical significance was set at p < 0.05. All statistical analyses were performed using GraphPad Prism 8 software (GraphPad Software, USA). The statistical significance of some experiments was confirmed using Student’s *t*-test, where: The statistical significance of some experiments was confirmed using Student's *t*-test, with appropriate symbols *, & or # where one or more symbols are used *, &, # for 0.01 ≤ p < 0.05; **, &&, ## for 0.001 ≤ p < 0.01; ***, &&&, ### for 0.0001 ≤ p < 0.001; ****, &&&&, #### for p < 0.0001.

## 3 Results

### 3.1 Hemolysate inhibits LPS-induced formation of neutrophil extracellular traps

**Figure 1:**
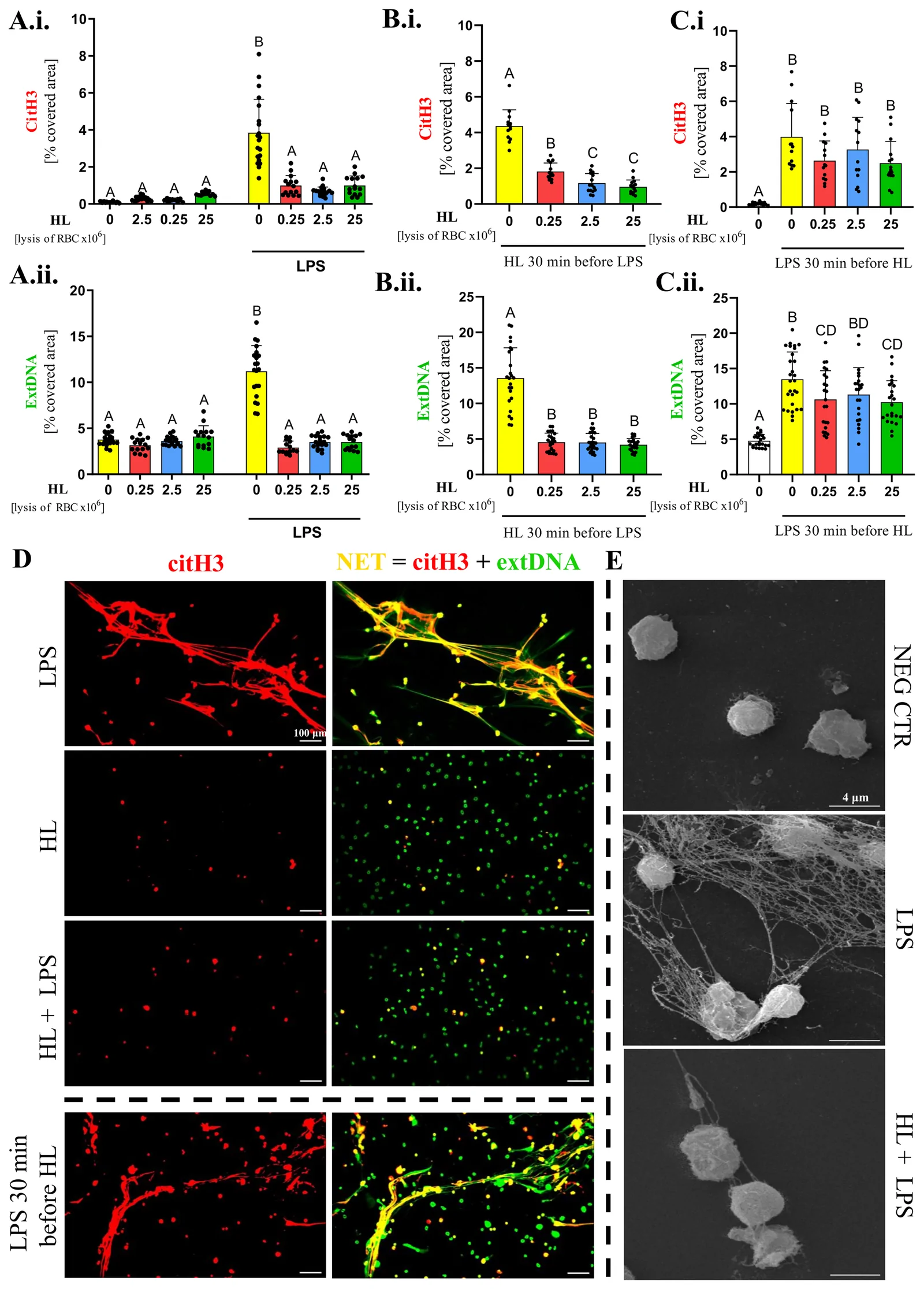
Effect of the hemolysate on the ability of mouse neutrophils to release neutrophil extracellular traps (NETs). Cells were incubated with hemolysate (HL) obtained from the lysis of 0.25×10^6^, 2.5×10^6^, or 25×10^6^ red blood cells (RBC) with or without lipopolysaccharide (LPS; 50 μg/ml) or left alone (0). NET formation was measured by the area of the FOV covered with extracellular DNA (extDNA) or citrullinated histone H3 (citH3) – marker proteins for NETs. **(A)** Neutrophils were incubated with the indicated concentrations of HL with or without LPS for 6 h, while **(B)** shows pretreatment (30 min) with HL before LPS and **(C)** shows pretreatment (30 min) with LPS before HL. The upper panel (A.i.-C.i.) presents quantification of citH3, while the middle panel (A.ii.-C.ii.) presents quantification of extDNA. **(D)** Representative images of neutrophils treated with LPS and/or HL, where green shows extDNA and red shows citH3 (to visualize NETs, images of both channels were overlaid; NET = extDNA + citH3). Scale bar = 100 μm. **(E)** Representative images of NETs obtained by scanning electron microscopy (SEM) where cells were treated with HL (2.5×10^6^ lysed RBC) and/or LPS or left untreated (NEG CTR). Scale bar = 4 μm. Results are expressed as mean values with SD (n ≥ 3), where n refers to the number of animals used to collect neutrophils. Within each experiment (cells from one mouse donor), multiple (4–5 on average) fields of view (FOVs) were imaged and analyzed; all raw data (for all FOVs) are presented as dots against the bars (mean value). Differences among groups were assessed by one-way ANOVA, with Bonferroni correction applied for *post hoc* comparisons. Statistical significance is indicated by different letters (p < 0.05).

NET release, estimated by quantifying its two overlapping components, extracellular DNA (extDNA) and citrullinated histones (citH3), using confocal microscopy, was induced by incubating neutrophils with lipopolysaccharide (LPS) but not with hemolysate (HL) alone at any of the 3 tested concentrations (**Figures 1A–D**). Moreover, when cells were exposed simultaneously to HL (regardless of its concentration) and LPS, or to HL 30 min before LPS, NET formation was completely suppressed (**Figures 1A, B, D**). NET release and its modulation by HL were further confirmed by scanning electron microscopy (SEM) (**Figure 1E**). In subsequent confocal microscopy evaluation, pre-treating neutrophils with LPS for 30 min before HL sustained NET formation (**Figure 1C**). A similar lack of NET inhibition was observed when HL was added 2 or 4 hours after LPS (**Supplementary Figure 2**). Since activation of p38 MAPK has been implicated in LPS-induced NET formation, changes in p38 phosphorylation were compared between HL-treated and untreated neutrophils (**Supplementary Figure 3**). It turned out that addition of HL prevented the phosphorylation of the investigated protein (**Supplementary Figures 3A, C**). As expected, p38 phosphorylation increased only following LPS stimulation, peaking at 15 and 30 min. Phosphorylation subsequently declined, as expected after 60 min. Importantly, this was not observed when cells were exposed to different NET stimuli as calcium ionophore (CI), where, unlike the LPS-treated group, phosphorylation was no longer reduced in the presence of hemolysate (**Supplementary Figure 3B**). Over time, phosphorylation gradually decreased, reflecting the progression of neutrophil activation toward NET formation. Crucially, HL was not cytotoxic toward neutrophils at the two lower concentrations. Of note, the color at the highest concentration (due to high hemoglobin levels) interfered with the viability measurement and prevented its determination with PrestoBlue assay (**Supplementary Figures 4A–D**). However, Annexin V/propidium iodide staining (**Supplementary Figure 5**) confirmed the above data to be valid for all hemolysates. While LPS itself was partially cytotoxic to neutrophils, inducing apoptosis (in approximately 25% of cells) and necrotic morphology in approximately 10%, hemolysates diminished this effect in a concentration-dependent manner. Whereas in 2D cultures LPS alone induced some cytotoxicity, neutrophils in 3D culture were protected from this effect. Hemolysate, if any, somewhat improved cell viability in the 3D system (**Supplementary Figure 4C**). To verify whether other neutrophil functions are also affected by HL, phagocytic capacity was tested. However, only minor changes were observed after treatment with the middle concentration of HL and/or LPS (**Supplementary Figure 6**).

### 3.2 The hemolysate components responsible for LPS-induced NET suppression, in order of magnitude, are hemoglobin, heme, and erythrocyte ghosts

**Figure 2:**
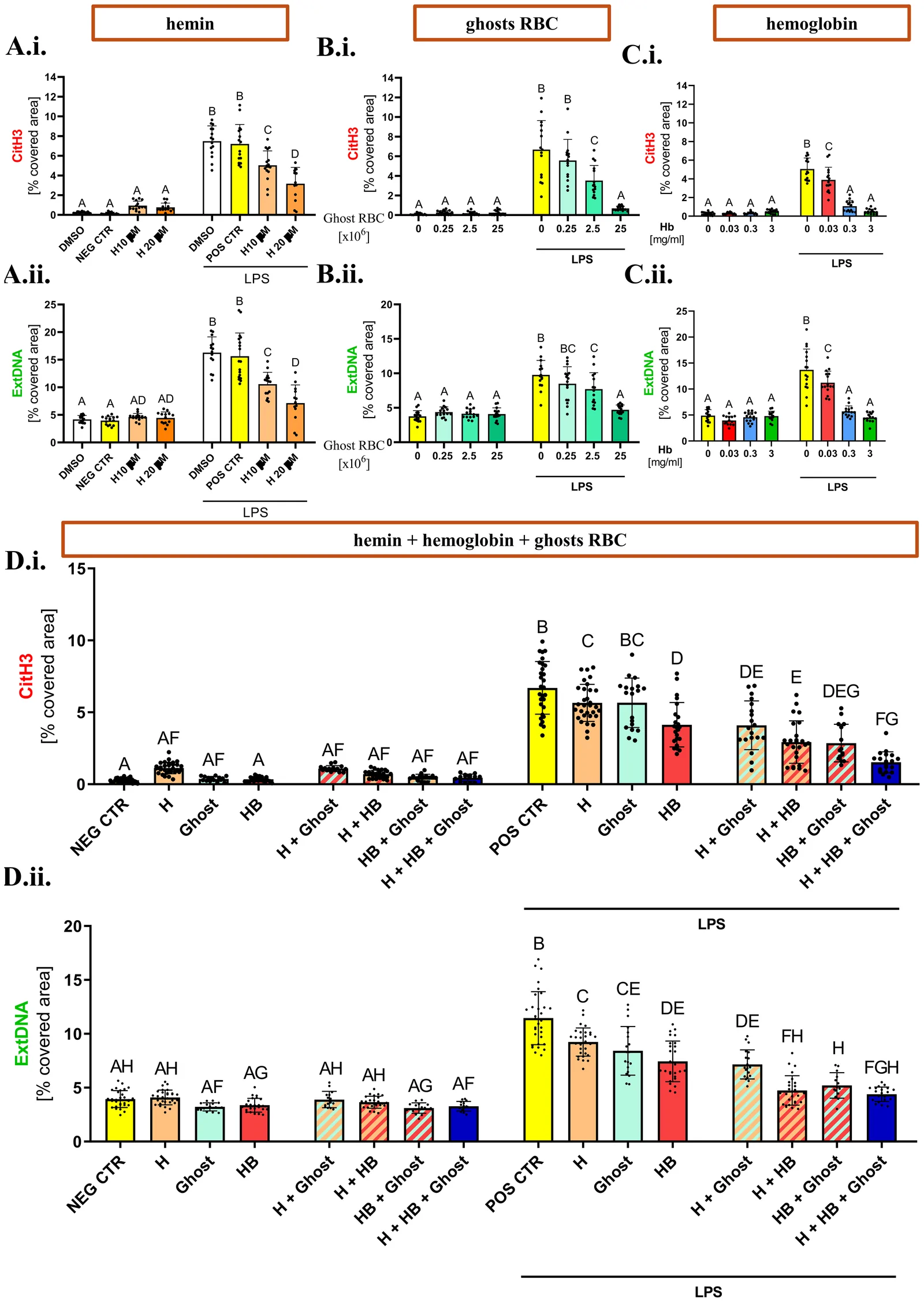
Effect of the components of hemolysate on NET formation. Neutrophils were exposed to **(A)** hemin at two concentrations (10 and 20 µM; H10/H20) alone or stimulated with lipopolysaccharide (LPS; 50 µg/ml/POS CTR) or left untreated (0/NEG CTR); **(B)** RBC ghosts (from lysis of 0.25×10^6^, 2.5×10^6^, or 25×10^6^ cells); or **(C)** hemoglobin (HB; concentrations: 0.03, 0.3, or 3 mg/ml), with or without LPS. **(D)** Mixed stimuli consisting of two or three of H, HB, and/or Ghost were added simultaneously with or without LPS. In all experimental setups, LPS incubation lasted 6 h. NET formation was measured by the area of FOV covered with extracellular DNA (extDNA) or citrullinated histone H3 (citH3), a marker protein for NETs. Graphs in the upper panel **(A.i.–D.i.)** represent analysis of citH3, and in the middle panel **(A.ii.–D.ii.)** of extDNA. Results are expressed as mean values with SD (n ≥ 3), where n refers to the number of animals used to collect neutrophils. Within each experiment (cells from one mouse donor), multiple (4–5 on average) fields of view (FOVs) were imaged and analyzed; all raw data (for all FOVs) are presented as dots against the bars (mean value). Differences among groups were assessed by one-way ANOVA, with Bonferroni correction applied for *post hoc* comparisons. Statistical significance is indicated by different letters (p < 0.05).

Based on our observations and the literature, we selected three components primarily present in HL - hemoglobin (HB), cell remnants (RBC ghosts), and heme – for more detailed scrutiny. Because hemolysate is a mixture of many compounds, some of which – such as HB – are proteins prone to auto-oxidation, we evaluated both HL and HB alone for changes in composition over time. To do this, we monitored changes in the absorbance spectrum after 6 hours (the incubation time for NET release) at 37°C. We know that oxyHB has its own specific absorbance peaks (around 540 and 570 nm) (40), as does metHB/heme (496 and 630 nm) (40–42). We detected both forms in the tested hemolysates (**Supplementary Figure 7**), and following the 6 h incubation, there was a drop in the Soret band [around 415 nm, characteristic of HB and heme components (40)] as well as in the peaks characteristic of oxyHB. This was in favor of the characteristic absorbance of metHB/heme (and other semi-products of HB disintegration), which rose around 480–520 nm and 590–650 nm (**Supplementary Figure 7**). To test the effect of heme, we used hemin (H) – structurally and functionally similar to heme but used in testing as an oxidized form (43, 44). Two different H concentrations (10 and 20 µM) produced weaker NET release by neutrophils exposed to LPS (**Figure 2A**), although this was much milder than that observed with full HL (**Figure 1A**). The observed effect in HL was independent of heme oxygenase 1 activity (**Supplementary Figure 8**). Further enrichment of hemin with HL (25×10^6^) led to complete suppression of NETs (**Supplementary Figure 9**). RBC ghosts (cell membranes with attached proteins) also contributed to NET suppression in a concentration-dependent manner, albeit to a lesser extent (**Figure 2B**). In addition, removing sialic acids from ghost RBC membranes or blocking the Siglec-E receptor did not cause reoccurrence of NETs (**Supplementary Figures 10**, **11**), as was the case in earlier studies in our team involving extracellular vesicles (6). Another, most abundant component, HB, also affected NET formation in a concentration-dependent manner (**Figure 2C**). However, only the lowest concentration of HB (0.03 mg/ml; corresponding to the concentration of HB in our lowest concentration of HL) only slightly suppressed NET formation (**Figure 2C**). A mixture of two of the three (H and Ghost, H and HB, or HB and Ghost) led to significant suppression of NETs (**Figure 2D**). Furthermore, a combination of all three components (by artificially recreating the mixture present in the hemolysate), i.e., ghost RBC (from lysis of 0.25×10^6^ cells), HB at the lowest concentration (0.03 mg/ml), and H at the lowest concentration (10 µM), resulted in a significant decrease in NET release by more than 50% (**Figure 2D**).

### 3.3 The effect of hemolysate on NET formation depends on the nature of the pro-inflammatory stimulus

**Figure 3:**
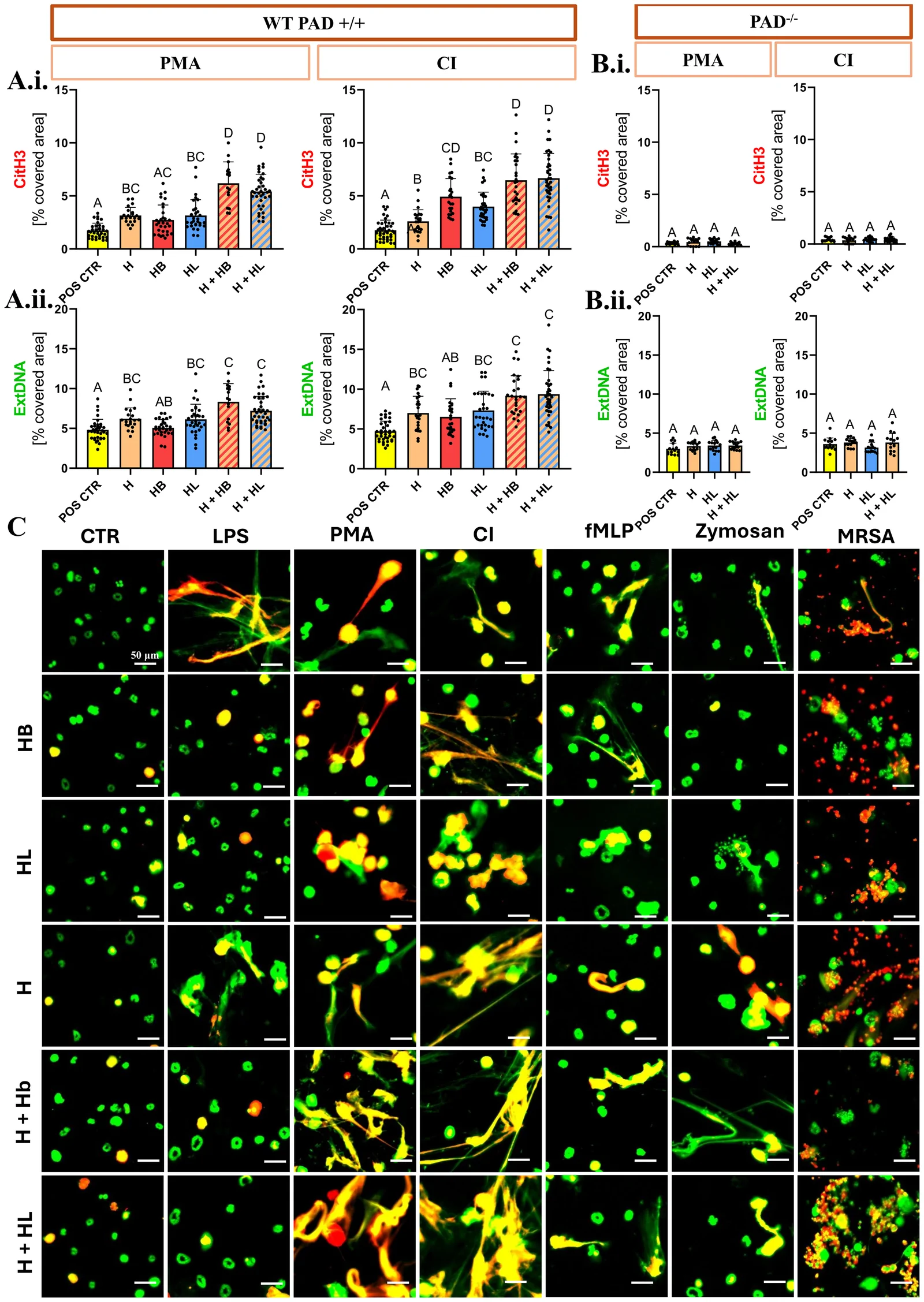
Quantification of NET formation after exposure to hemolysate and/or its components with multiple proinflammatory stimulants. Neutrophils were isolated from the bone marrow of WT (PAD^+/+^) mice and PAD^-/-^ knockout mice. All experiments in this configuration were performed in RPMI 1640 medium. Cells were exposed to hemoglobin (HB; 0.3 mg/ml), hemin (H; 10 µM), or hemolysate (HL from lysis of 2.5×10^6^ red blood cells), either alone or in combination (H+HL/H+HB). In addition, neutrophils were treated (POS CTR) with lipopolysaccharide (LPS; 50 μg/ml), PMA (100 nM), calcium ionophore (CI; 10 µM), fMLP (1 µM), Zymosan (100 µg/ml), or MRSA (10:1 bacteria to cells). **(A)** Quantification of NETs formed by neutrophils from WT mice treated with CI/PMA. NETs were stained, and FOVs were quantified for the area of (A.i.) citrullinated histone (citH3) and (A.ii.) extracellular DNA (extDNA). **(B)** Neutrophils were treated with H, HL, or both, and with CI/PMA. Below **(C)** are representative images of all combinations of HL and/or its components and all stimuli or left untreated (CTR). Merged channels were used to visualize NETs (NET = green extDNA + red citH3). Scale bar = 50 μm. Results are expressed as mean values with SD (n ≥ 3). Results are expressed as mean values with SD (n ≥ 3), where n refers to the number of animals used to collect neutrophils. Within each experiment (cells from one mouse donor), multiple (4–5 on average) fields of view (FOVs) were imaged and analyzed; all raw data (for all FOVs) are presented as dots against the bars (mean value). Differences among groups were assessed by a Kruskal–Wallis test followed by Dunn’s multiple-comparison test for *post hoc* comparisons. Statistical significance is indicated by different letters (p < 0.05).

To date, we have tested only the impact of HL on LPS- induced NETs. Therefore, we tested whether HL has a universal effect when NETs are induced by other known biological and pharmacological inducers. Among them, we tested PMA, CI, fMLP, Zymosan, and MRSA. PMA is a well-known plant-based compound that induces NETs through a NOX- dependent pathway (45, 46). By contrast, we also tested CI as another NET inducer that acts independently of the NOX pathway (46, 47); the strong PMN chemoattractant fMLP, which activates many proinflammatory signaling pathways (48); zymosan (a *Saccharomyces cerevisiae* cell wall component); and the bacterium MRSA. The stimuli with the most noticeable NET formation, apart from LPS, were calcium ionophore (CI) and PMA (**Figure 3**; **Supplementary Figure 12**). We evaluated the influence of all of these stimuli together with not only H (10 µM), HB (0.3 mg/ml), and HL (2.5 × 10^6^), but also combinations of H with HB or H and HL at the same concentrations. Not only did the hemolysate and its main components, taken separately, show no inhibition of PMA/CI induced NETs, but we also observed a tendency toward increased NET production (**Figure 3A**). When cells were exposed to CI/PMA together with a mixture of H and HB or HL, the observed effect was even stronger (**Figure 3A**). This stands in clear contrast to when cells were stimulated with LPS and a mixture of HL or its components (**Figure 2D**). Zymosan, fMLP, and MRSA did not show a change in the amount of NET after treatment with HL and/or its components (**Figure 3C**; **Supplementary Figure 12**) yet due to the fact that zymosan and MRSA showed nonspecific fluorescence signal, only limited quantitative data could be generated (**Supplementary Figure 12**). To verify whether the observed increase is in fact associated with NET formation rather than caused by cell death (e.g., NETosis), we tested the two most potent reagents, PMA/CI, in the same setting, but limited to H and HL and their combination, in mice with PAD4 knockout whose neutrophils do not release NETs (**Figure 3B**). This resulted in no effect in any case. This indeed showed us that all of them depended on PAD4-mediated NET release. Overall, although the components themselves or HL as a whole do not have a significant effect on NET release, HL may behave differently depending on the inflammatory response.

### 3.4 Hemoglobin contributes to NET inhibition, potentially through LPS binding

**Figure 4:**
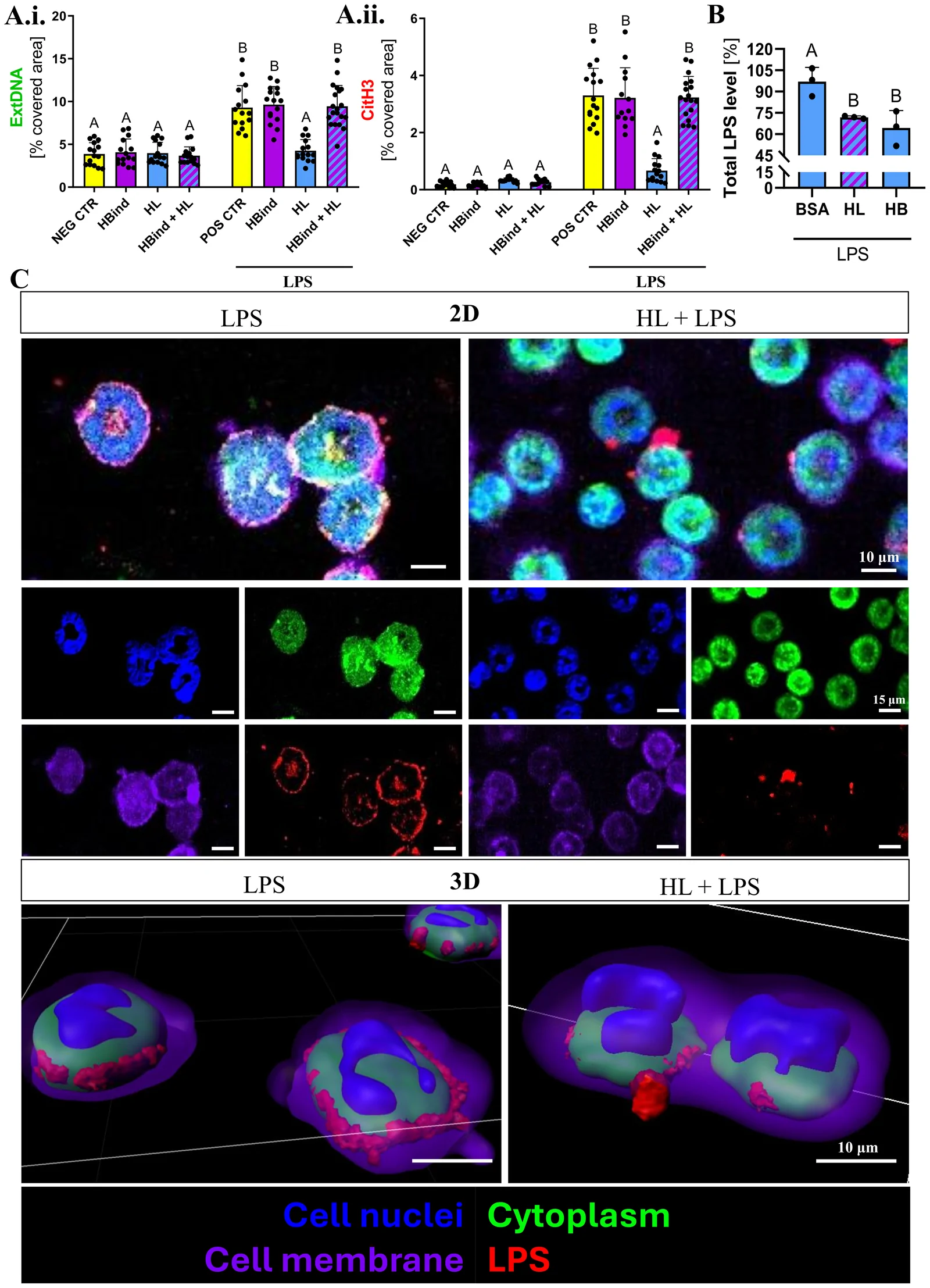
Evaluation of the role of hemoglobin in NET suppression. Hemoglobin (HB) and ghosts were depleted from hemolysate using HemogloBind (HBind) and high-speed centrifugation, respectively, leaving the other components. NETs were stained with citrullinated histone (citH3) and extracellular DNA (extDNA), and the area of fluorescence was quantified. Cells where stimulated with lipopolysaccharide (LPS, POS CTR) at a concentration of 50 μg/ml or left untreated (NEG CTR). **(A)** Quantification of HB-depleted hemolysate (HL, from lysis of 2.5×10^6^ red blood cells). NET formation was quantified by the area of the FOV covered by extracellular DNA (extDNA). The graphs (A.i.) at the top show results without additional LPS treatment, and the graph (A.ii.) below shows results with additional LPS treatment. **(B)** Quantification of LPS levels in mixtures of BSA, HL, or HB after 1 h incubation, measured with the fluorescence rFC assay. Below **(C)** are representative images of neutrophils incubated with fluorochrome-labeled lipopolysaccharide (AlexaFluor 568, red color; 15 μg/ml). Nuclei of PMN were stained with HOECHST (blue), cytoplasm with CellTracker (green), and labeled with Ly6G (AF647; violet). Upper panel **(C)** shows images of labeled LPS binding to the cell surface in 2D (upper panel) and 3D reconstruction (lower panel), whereas the left side shows images of cells incubated only with LPS, and the right side shows LPS with HL. Scale bar = 10 μm for upper 2D pictures and 3D, 15 μm for lower 2D pictures. Results are expressed as mean values with SD (n ≥ 3), where n refers to the number of animals used to collect neutrophils. Within each experiment (cells from one mouse donor), multiple (4–5 on average) fields of view (FOVs) were imaged and analyzed; all raw data (for all FOVs) are presented as dots against the bars (mean value). Differences among groups were assessed by one-way ANOVA, with Bonferroni correction applied for *post hoc* comparisons. Statistical significance is indicated by different letters (p < 0.05).

Given that the observed effect was specific to LPS (**Figures 1**–**3**), we concluded that it was likely due to a mechanistic effect rather than activation of internal mechanisms, especially since inhibition did not occur when the HL was present after LPS (**Figure 1C**). Since the observed relationship was clearly dependent on HB and less on the other two components of HL (**Figure 2**), we decided to focus on it. To verify whether HB present in the hemolysate is indeed co-responsible for NET inhibition in the presence of LPS, we removed HB from the HL using HemogloBind (HBind) and stimulated the cells with the mixture of the remaining HL components. Indeed, after HB removal, the level of NETs released by neutrophils markedly increased (**Figure 4A**; **Supplementary Figure 13**), confirming that this molecule in HL is responsible for NET inhibition. The reagent (HBind) used to remove HB did not interfere with NET formation. Similarly, HB was depleted from HL using zinc chloride, yielding similar results (**Supplementary Figure 14**). Furthermore, cell viability did not change when HB was depleted from HL (**Supplementary Figure 15**). In addition, we assessed LPS levels upon addition of LPS to HL (with an rFC assay after 1 hour of co-incubation). LPS levels were significantly decreased after co-incubation with HB or HL (**Figure 4B**). Moreover, we used fluorochrome-labeled LPS and demonstrated that, following incubation with the HL, less LPS was internalized or bound to neutrophils (**Figure 4C**).

### 3.5 Very high concentrations of LPS partially reverse the inhibitory effect of hemoglobin/hemolysate

**Figure 5:**
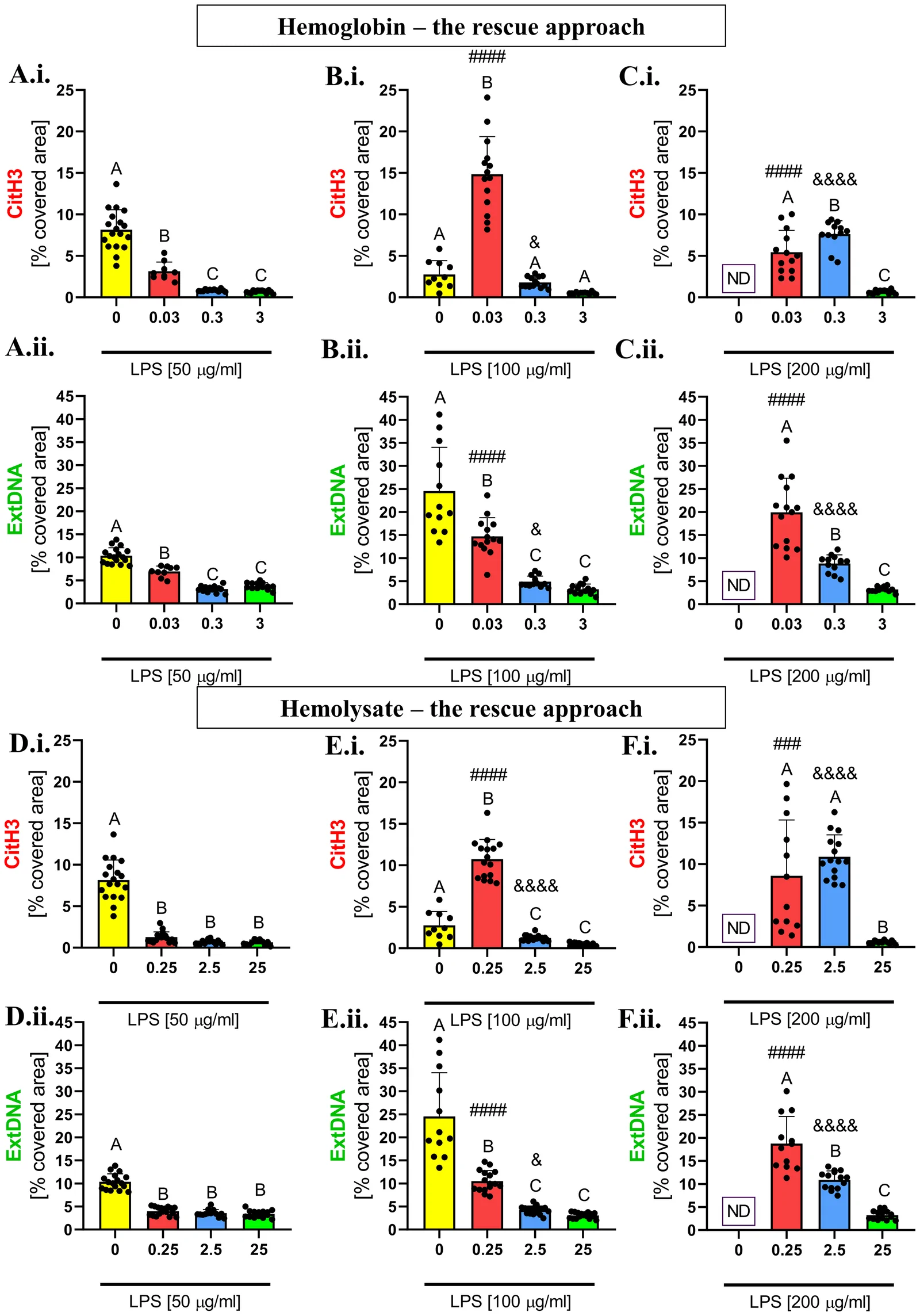
Effects of high LPS concentration on hemolysate ability to inhibit NET formation (the rescue approach). **(A–C)** Cells were incubated with hemoglobin (HB; 0.03, 0.3 or 3 mg/ml) or **(D–F)** hemolysate (HL) obtained from the lysis of 0.25×10^6^, 2.5×10^6^ or 25×10^6^ red blood cells (RBC). Additionally, neutrophils were treated with LPS at concentrations of 50, 100 or 200 μg/ml or left untreated (0). In all experimental setups, LPS incubation lasted 6 h. NETs were stained, and FOVs were quantified for the area of **(A.i. – F.i.)** citrullinated histone (citH3) and **(A.ii. – F.ii.)** extracellular DNA (extDNA). ND, signal not detected. Results are expressed as mean values with SD (n ≥ 2), where n refers to the number of animals used to collect neutrophils. Within each experiment (cells from one mouse donor), multiple (4–8 on average) fields of view (FOVs) were imaged and analyzed; all raw data (for all FOVs) are presented as dots against the bars (mean value). Differences among groups were assessed by one-way ANOVA, with Bonferroni correction applied for *post hoc* comparisons. Statistical significance is indicated by different letters (p < 0.05). Additionally, to identify the groups in which neutrophils with higher concentrations of LPS, despite the presence of HL, produced NETs, we used a t-test; # or & indicates a statistically significant difference compared with the LPS 50 μg/ml-treated cells with 0.25×10^6^ or 2.5×10^6^, respectively. & indicate p < 0.05, ### for p < 0.001 and #### or &&&& indicate p < 0.0001.

All tested concentrations of HB and HL inhibited NET formation in the presence of 50 µg/ml LPS (**Figures 5A, D**; **Supplementary Figure 16**), and we showed that this is because at least some LPS is trapped by hemoglobin (**Figure 4**). Because of this, we performed experiments aimed at reversing this phenotype using very high concentrations of LPS (100 and 200 µg/ml) and the full range of HL and HB concentrations. Both of them alone caused neutrophil death (only extDNA was released; low to non citH3 was detected) (**Figures 5B, C, E, F**; **Supplementary Figure 16**). However, at the lowest HB (0.03 mg/ml) and HL (0.25×10^6^) concentrations, neutrophils not only did not die but also released as many NETs as when treated with 50 µg/ml LPS alone (**Figures 5B, E**; **Supplementary Figure 16**). At 200 µg/ml of LPS, the next higher concentrations of HB (0.3 mg/ml) and HL (2.5×10^6^) also blocked neutrophil death and restored the capacity to release NETs whereas the lowest ones failed to fully protect the cells against the highest LPS concentration, causing cell death to increase again, while still retaining a partial protective effect (**Figures 5C, F**, **Supplementary Figure 16**).

### 3.6 Hemolysate derived from red blood cells of endotoxemic mice exhibits reduced NET-suppressive activity

**Figure 6:**
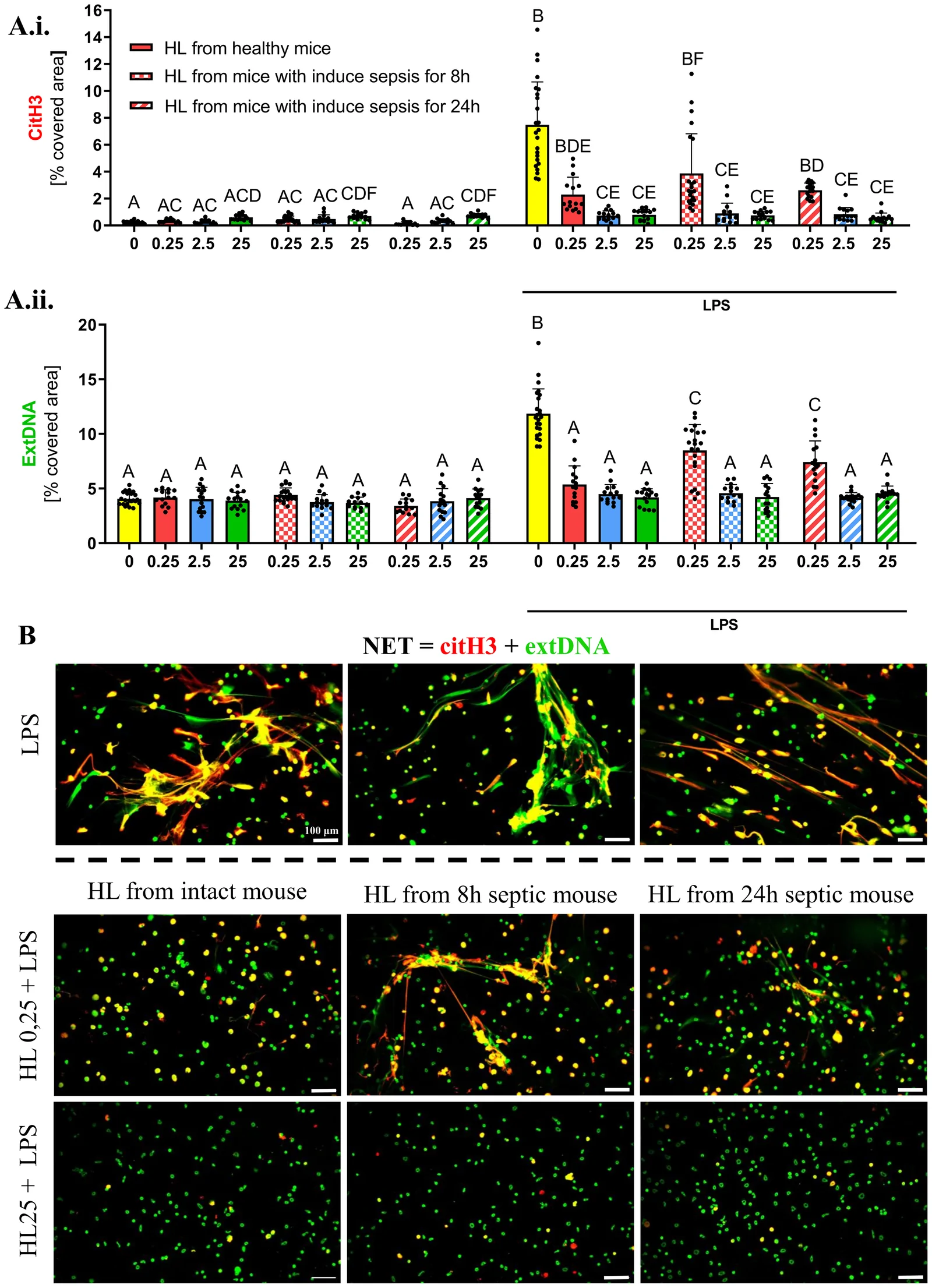
Effect of recent endotoxemia on the ability of hemolysate to inhibit NET formation. Cells were incubated with hemolysate (HL) obtained from the lysis of 0.25×10^6^, 2.5×10^6^, or 25×10^6^ red blood cells (RBC). HL was obtained from intact or endotoxemic mice (lipopolysaccharide (LPS) treatment at 1 mg/kg body weight for 8 h or 24 h). Additionally, neutrophils were treated with LPS at 50 μg/ml or left untreated (0). NETs were stained, and FOVs were quantified for the area of **(A.i.)** citrullinated histone (citH3) and **(A.ii.)** extracellular DNA (extDNA). **(B)** Representative images of neutrophils treated with LPS and/or HL, where green shows extDNA and red shows citH3 (to visualize NET, images of both channels were overlaid; NET = extDNA + citH3). Scale bar = 100 μm. Results are expressed as mean values with SD (n ≥ 3), where n refers to the number of animals used to collect neutrophils. Within each experiment (cells from one mouse donor), multiple (4–5 on average) fields of view (FOVs) were imaged and analyzed; all raw data (for all FOVs) are presented as dots against the bars (mean value). Differences among groups were assessed by one-way ANOVA, with Bonferroni correction applied for *post hoc* comparisons. Statistical significance is indicated by different letters (p < 0.05).

Until now, HL had always been obtained from healthy mice. Therefore, we asked whether a similar inhibitory effect on NETs could also be achieved when HL was derived from mice with ongoing endotoxemia (for 8 or 24 hours), thereby inducing systemic inflammation via LPS. During systemic inflammation, various changes occur in the organism, including those affecting RBC (49). In general, NET suppression was still observed, but not at the lowest HL concentration (when the HL was obtained from 0.25×10^6^ RBCs) (**Figure 6**). HL obtained from endotoxemic mice showed no cytotoxicity to cells (**Supplementary Figure 4D**). As with ghost RBCs from healthy mice, inhibition of NETs was independent of sialic acid or Siglec-E (**Supplementary Figure 11**).

### 3.7 *In vivo* hemolysis may be associated with attenuated neutrophil inflammatory response to LPS due to reduced NET formation

**Figure 7:**
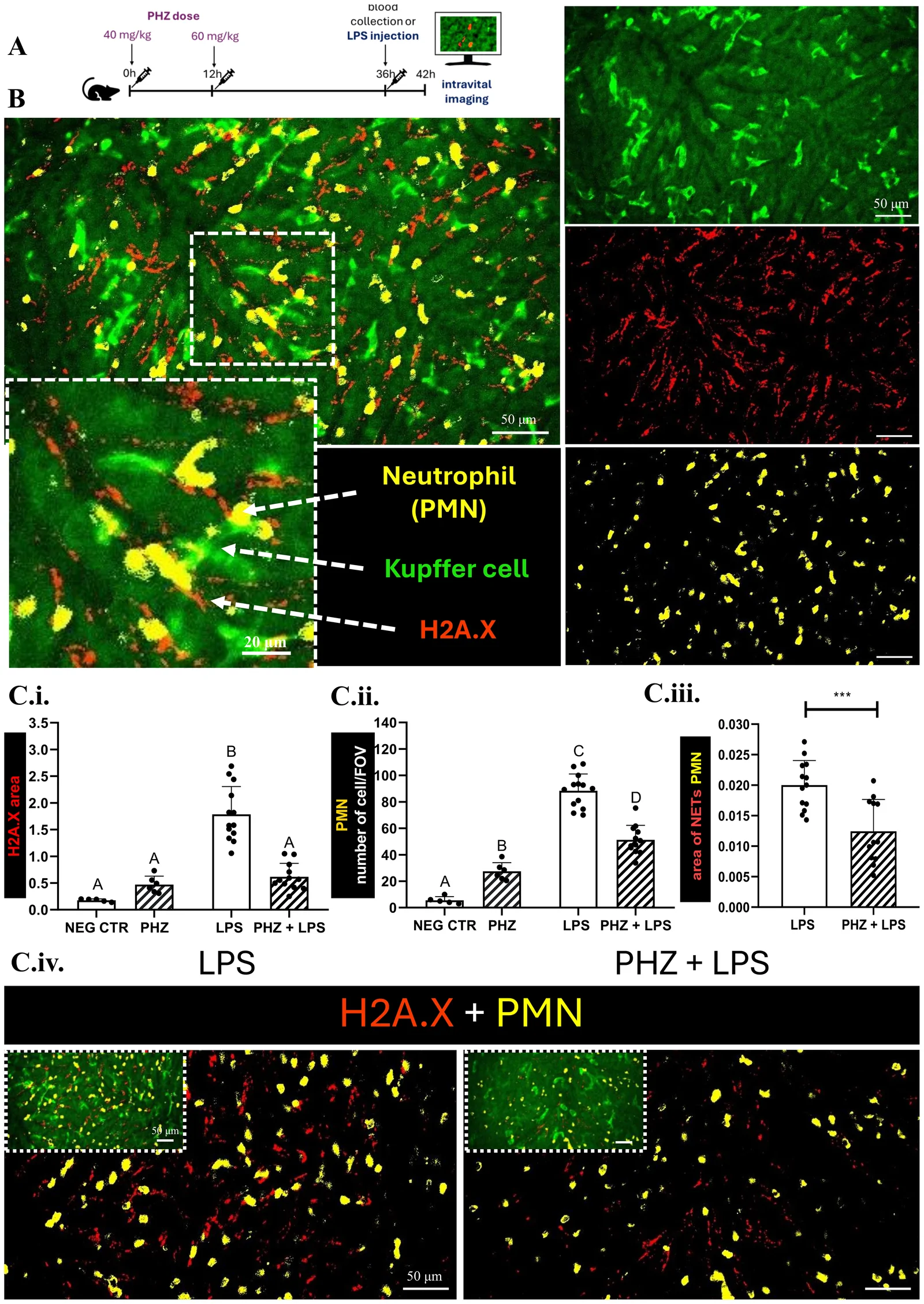
Observation of endotoxemia induced in hemolytic mice. C57BL/6J mice were treated with phenylohydrazine (PHZ) to induce hemolysis. **(A)** PHZ was injected intraperitoneally at time 0 (40 mg/kg body weight; b.w.) and again after 12 h (60 mg/kg b.w.). Mice were left for 24 h, and lipopolysaccharide (LPS; 1 mg/kg b.w.) was then injected intravenously for 6 h. **(B)** The panel shows the liver of mice treated with LPS alone, where dark green color is the autofluorescence of hepatocytes, light green fluorescence is from F4/80-stained Kupffer cells (AF488), false yellow color indicates Ly6G^+^ neutrophils (originally BV421), and false red color indicates H2A.X histones (originally AF647) – a marker of NETs. Separate channels are presented on the left side of the panel. Quantification of **(C.i.)** the area of H2A.X histone and **(C.ii.)** neutrophils per FOV for mice treated with LPS, PHZ, PHZ + LPS, or left untreated (NEG CTR) was assessed. **(C.iii.)** The H2A.X/PMN ratio was also calculated. **(C.iv.)** Representative images of histone H2A.X area for LPS-treated experimental groups. Scale bar = 50 μm. Results are expressed as mean values with SD (n ≥ 3) for LPS-treated mice. In each mouse, multiple (4–5 on average) fields of view (FOVs) were imaged and analyzed; all raw data (for all FOVs) are presented as dots against the bars (mean value). Statistical differences between groups were tested using Student’s t-test, where *** denotes 0.0001 ≤ p < 0.001.

To investigate whether the observed phenomenon also occurs in living organisms, we employed intravital microscopy. To induce hemolysis, mice were treated with phenylhydrazine (PHZ), followed by endotoxemia induced by intravenous LPS injection lasting 6 hours (**Figures 7A–C**; **Supplementary Figure 17**). Both the level of histone H2A.X indicative of NETs (**Figure 7C.i.**) and the number of neutrophils that migrated to the liver (**Figure 7C.ii**.) were compared between mice with and without hemolysis prior to LPS stimulation. Indeed, neutrophil infiltration and NET release were significantly decreased in the former group. The ratio of NETs to neutrophils further confirmed that these cells released much less NETs (**Figure 7C.iii**).

## 4 Discussion

A variety of diseases and pathophysiological conditions lead to uncontrolled red blood cell (RBC) lysis in the circulation. These include inherited disorders such as sickle cell anemia (SCA) and hereditary spherocytosis, autoimmune disorders, and acquired factors, including infections, toxins, and mechanical damage (e.g., associated with prosthetic heart valves) (8–10, 12, 50–53). Hemolysis may also occur in sepsis, a life-threatening organ dysfunction that originates from a dysregulated immune response (12, 54). Some studies have shown higher mortality in sepsis among patients with higher cell-free hemoglobin (CFH) and lower hemopexin (Hx; heme scavenger) or haptoglobin (Hp; hemoglobin scavenger) levels (29, 55–58). Others find no strong correlation between disease severity (as measured by Sequential Organ Failure Assessment; SOFA) and hemolysis (57, 59). It seems that there is still a lack of studies or meta-analyses that would allow for a clear determination of the impact of hemolysis on systemic inflammation (57). On the other hand, there is excessive activation of the immune system during sepsis, particularly of neutrophils, with increased recruitment and NET production. Accumulation of NETs during an exaggerated inflammatory response is associated with complications such as vaso-occlusive crises (particularly in sickle cell disease [SCD]) (7, 19) as well as acute respiratory distress syndrome and the production of anti-neutrophil cytoplasmic antibodies (ANCA) (60). In fact, extracellular histones are among the major causes of death in sepsis (61). Furthermore, NETs persisting in the vasculature may undergo self-renewal, with consequences for subsequent infections (39).

Given that during systemic inflammation, NET formation occurs in the blood, where neutrophils are exposed not only to the inflammatory stimulus but also to hemolysate, we determined the impact of LPS, the most immunogenic component of Gram-negative bacteria, which are responsible for 45-50% of all sepsis cases (versus 35-45% for Gram-positive bacteria) (62). Moreover, we did not focus on a single hemolysate component but studied it as a whole and tested it at 3 different concentrations, reflecting varying degrees of RBC lysis. Although the LPS concentrations used in this study are higher than those typically reported in patients with sepsis, they were selected considering the LPS sensitivity of murine neutrophils and the requirements of *ex vivo* mechanistic models (63). Applied LPS concentrations do not directly correspond to the levels of biologically active endotoxin encountered *in vivo* due to the fact that after entering the circulation, LPS rapidly interacts with plasma proteins and lipoproteins and undergoes clearance or internalization by immune cells and the liver, substantially influencing the fraction available to activate target cells (64). Concentrations similar to the one we used have been widely reported in studies investigating LPS-induced neutrophils activation and NET formation to obtain robust and reproducible responses (36, 37, 65, 66).

Red blood cells contain mostly hemoglobin (HB) by volume, but also carry glucose for energy, required enzymes, ions, and other molecules (10, 67). The cell membrane, along with the attached proteins and glycoproteins, also plays an important role in the bloodstream and in cell interactions (68, 69). Moreover, free hemoglobin eventually releases heme, and membrane ghosts (cell membrane remains) also circulate in the blood. Of the three, numerous studies have highlighted heme’s proinflammatory effects. Ohbuchi et al. showed a dose-dependent increase in NET production by human neutrophils after hemin treatment (70). However, in these analyses, only the amount of extracellular DNA was measured quantitatively. Another group reported that TNF-α–primed neutrophils generated NETs in the presence of hemin, but not hemoglobin (19). Therefore, neutrophils were already in a pro-inflammatory state when hemin was added, which might explain the discrepancy with our data, as we treated resting cells simultaneously with LPS and hemin. It was shown previously that cell sensitivity to heme depends on its pre-activation (71–73).

We hypothesized that NET production could be affected by erythrocyte content, given that all hemolysate components are classified as DAMPs. We further hypothesized that RBC DAMPs would increase NET release, acting in a pro-inflammatory manner, probably much more profoundly than heme/hemin itself. Surprisingly, however, even the lowest HL concentration completely inhibited NETs. We selected phosphorylated p-38 MAPK protein (p-p38 MAPK) as an early marker of neutrophil activation (74, 75). Similarly, HL markedly attenuated LPS-induced neutrophil activation, whereas it did not exert a comparable inhibitory effect in cells stimulated with the alternative pro-inflammatory stimulus, calcium ionophore (CI). Furthermore, the decline in p-p38 MAPK levels at 60-minute time points observed in the LPS alone and CI + HL groups suggests the completion of the early signaling phase and progression toward the downstream effector events leading to NET formation (76, 77). Notably, the presence of HL appeared to accelerate this transition in CI-stimulated neutrophils, as reflected by the earlier (30 min) decline in p-p38 MAPK levels.

Focusing on the mouse setting, we hence examined each of its major components separately (HB, heme, ghosts), and each inhibited LPS-induced NETs in a concentration-dependent manner, with HB > hemin > ghosts. Guided by our findings, we subsequently artificially reconstructed the hemolysate content using the lowest inhibitory concentrations of the three components studied, thereby achieving a cumulative effect similar to that of HL. Already, pairs of the three components synergistically reduced NET release more than when applied separately, but when all were applied simultaneously, the effect was most pronounced.

In contrast to the pro-NET reports cited above, some articles describe hemin’s anti-inflammatory roles. For example, in sepsis models, hemin has been shown to reduce the pro-inflammatory response by inducing HO-1 or CO (78–80). i.e., by initiating the anti-inflammatory, cytoprotective pathway. However, in our model, the HL effects were independent of HO-1. Hemin binds directly to Toll like receptor 4 (TLR4) via MD2, acting as its agonist (81, 82), although it may also be transported through the cell membrane and thus initiate a cellular response more directly. In general, reports on the anti-inflammatory role of hemin are rare.

Ghost RBCs are remnants of the cell membrane. There is growing interest in them as drug delivery vehicles due to their unique properties (83, 84). They are considered immunologically silent because they consist mainly of elements (membrane and surface receptors/proteins) exposed to the extracellular milieu, although they lack internal contents. In our previous studies, we observed that erythrocyte-derived extracellular vesicles suppress NETs via Siglec-E interactions (6). Moreover, whole erythrocytes exhibited a similar pattern (36). The RBC membrane has the most numerous ligands for this receptor: sialic acids (85). Thus, we employed two approaches to neutralize this binding (a Siglec-E inhibitor and sialidase to remove its ligand), but neither restored NETs, ruling them out as the anti-inflammatory entities herein. Among other options, ghost RBCs tend to externalize phosphatidylserine (PS), which promotes phagocytosis (83), and PS has also been shown to enhance anti-inflammatory effects in macrophages (86).

The most surprising finding was that HB itself was not pro-inflammatory; rather, the proinflammatory effects stem from the heme and iron it contains. Despite this, the presence of free HB in the bloodstream has consequences. Vats et al., using intravital microscopy, clearly demonstrated that mice with SCD, when systemically challenged with oxyHB, exhibited increased NET production and pulmonary vaso-occlusion. However, control healthy mice challenged with oxyHB released only rare spontaneous NETs (87). Cell-free HB, if not scavenged, may bind NO (which can lead to various vascular pathologies) and be autoxidized (88). This can cause HB to break down into a dimer, then into metHB, ultimately releasing heme (89). While not strongly proinflammatory on its own, HB significantly increased ROS production and pro-inflammatory cytokine levels in neutrophils, monocytes, and macrophages in the presence of lipoteichoic acid (LTA) (90, 91), and even enhanced LPS-induced mortality in mice, although the latter was assessed with much higher doses of both LPS and HB (both by up to 30-fold) (92). Hemolysis occurring before infection may also polarize macrophages toward a more anti-inflammatory phenotype that clears damaged RBCs or Hp-HB complexes (93, 94).

It is worth noting that the inhibition observed in our study occurred when cells were treated with LPS and HL simultaneously or when HL was added before LPS. But our *ex vivo* system contained only neutrophils. Furthermore, when cells were stimulated by other factors that lack direct biological counterparts in sepsis, such as PMA or the calcium ionophore (CI), HL exhibited a strong proinflammatory increase in NET formation. Importantly, LPS and PMA *versus* CI act through different pathways (NOX-dependent or NOX-independent, respectively), to induce NET release, but all of them depend on PAD4 (95), which we confirmed in PAD4-deficient mice. These observations led us to examine HL and its components from a more mechanistic perspective. When we removed HB from HL by either of the two approaches, NET inhibition was absent. We should underline that the precipitation with ZnCl_2_ removed HB non-specifically, along with other proteins. The HemogloBind method, unlike the ZnCl_2_ method, removed HB specifically but also removed the ghosts (due to the required high-speed centrifugation), which further confirms its rather low anti-inflammatory potential. The available literature indicates that HB has multiple LPS-binding sites (96). Other studies show that HB binds not only to LPS but also to LTA (97). In line with this, isolated HB peptides (B59) were also shown to bind to bacteria (*E. coli* and *P. aeruginosa*; both Gram-negative) (96), but MRSA carrying LTA did not alter NET release in our system. The latter might be due to fewer LTA-binding sites. In contrast, HB effects were indeed driven by LPS sequestration, as shown by estimates of free LPS levels and their availability to bind to neutrophils. Moreover, when we oversaturated neutrophils with LPS (to the point it caused their death), and then added hemolysate or hemoglobin, the phenotype was rescued: neutrophils were viable and effectively produced NETs. This underlines that hemolysate containing hemoglobin might, in fact, play a positive role in vascular inflammation, minimizing cytotoxicity (and even mortality) by trapping excess LPS.

What surprised us was that, although the HL strongly inhibited the NETs, the HL obtained from erythrocytes collected 8 hours into endotoxemia did not inhibit NETs at the lowest concentration. RBCs can accumulate cytokines and chemokines (e.g., TNF-α, IL-1β, CXCL10) (49). Moreover, during sepsis, RBCs undergo multiple changes such as increased intracellular Ca^2+^ levels (98), decreased antioxidant production (99), and morphological changes in and out of the cell: RBC membrane deformations (100) and decreased sialic acids (36, 101). Additionally, HB may become oxidized (102) leading to denaturation/morphological changes of HB and formation of its aggregates such as Heinz bodies (103, 104) and band-3-bound hemichromes (105). The formation of HB aggregates alone could reduce the number of available LPS-binding sites, and collectively, the above changes may have contributed to impaired NET inhibition by septic HL.

Critically, we confirmed that the observed phenomenon also operates in *in vivo* during PHZ-induced hemolysis in mice, as verified using intravital microscopy. In mice, we observed that fewer neutrophils migrated into the hepatic sinusoids during endotoxemia, resulting in fewer NETs being released. Pfefferlé et al. demonstrated that heme signaling inhibited the pro-inflammatory activation of macrophages, thereby protecting the liver from damage due to sterile inflammation (106). Although anti-CD40 antibodies were used to induce inflammation, a decrease in pro-inflammatory factors and liver damage was observed (106). A similar effect was detected after administering both phenylhydrazine (PHZ) and heme conjugated with albumin. Moreover, there was a decrease in the mRNA levels of pro-inflammatory cytokines and chemokines in macrophages primed with heme-albumin and subsequently with LPS, compared to those primed with LPS alone (94). Another team also reported impaired innate immune responses following *Klebsiella pneumoniae* (Gram-negative) infection in a model involving hemolysis induced by transfusion of stressed RBCs. The effect was similarly immunosuppressive and associated with nuclear factor erythroid 2-related factor 2 (Nrf2) activation (94, 107). This was related to the polarization of macrophages toward erythrophagocytosis.

Our findings do not call into question previous observations regarding the role of hemolysis; however, they shed new light on related research and on the impact of hemolysate on NET release. We postulate that hemolysis, as a primary event preceding systemic inflammation, may have several consequences, including masking the initial symptoms of LPS-induced sepsis caused by Gram-negative bacteria, such as NET release. In fact, early NET inhibition might not be desirable, as NETs initially entrap bacteria (7). Alternatively, hemolysis *in vivo* may impair the pro-inflammatory response by depleting cells that mediate it. We have just shown that intact erythrocytes can inhibit NET formation, but not those collected from septic individuals (36). It is also likely that cell migration occurs earlier after hemolysis induction, leading to immune exhaustion.

Unfortunately, hemolysis during sepsis is sometimes overlooked because there is no optimized method for detecting it. Some meta-analyses report markedly increased serum inflammatory factor concentrations in sepsis caused by gram-negative bacteria, with greater increases in disease severity compared with gram-positive sepsis (108), whereas others show lower mortality in gram-negative sepsis (109). Further investigation into the correlation between Gram-negative or Gram-positive bacterial sepsis and hemolysis is warranted.

Many papers report a correlation between sepsis severity and patient mortality but do not provide a clear explanation for it or distinguish between sepsis induced by different pathogens (26, 29). In line with this, there is no consensus on whether hemolysis is primarily a biomarker of severe disease, a direct driver of organ injury, or both. Moreover, hemolysis is sometimes not accompanied by marked anemia (57). However, its mechanistic role remains the most obscure: is it good that it occurs, or is it bad? Whereas the latter answer is generally considered correct, our study shows that both answers may be correct, but depending on the type of bacteria causing sepsis. During Gram-negative bacteria-driven inflammation, free hemoglobin might scavenge LPS (which is commonly shed (110)), preventing cytotoxicity and overwhelming NET formation. But the question remains open for Gram-positive and polymicrobial sepsis, and thus far published reports do not differentiate between them.

## Data Availability

The original contributions presented in the study are included in the article/**Supplementary Material**. Further inquiries can be directed to the corresponding author.
