# Supplementary material for: One trigger, two fates: hemolysate shapes neutrophil extracellular traps (NETs) formation in a context-dependent manner
Source: Front Immunol. 2026 Sep 3;17:1917185. doi: 10.3389/fimmu.2026.1917185 (PMC13581507; doi:10.3389/fimmu.2026.1917185)
Supplement: Supplementary file 1 [file DataSheet1.pdf]

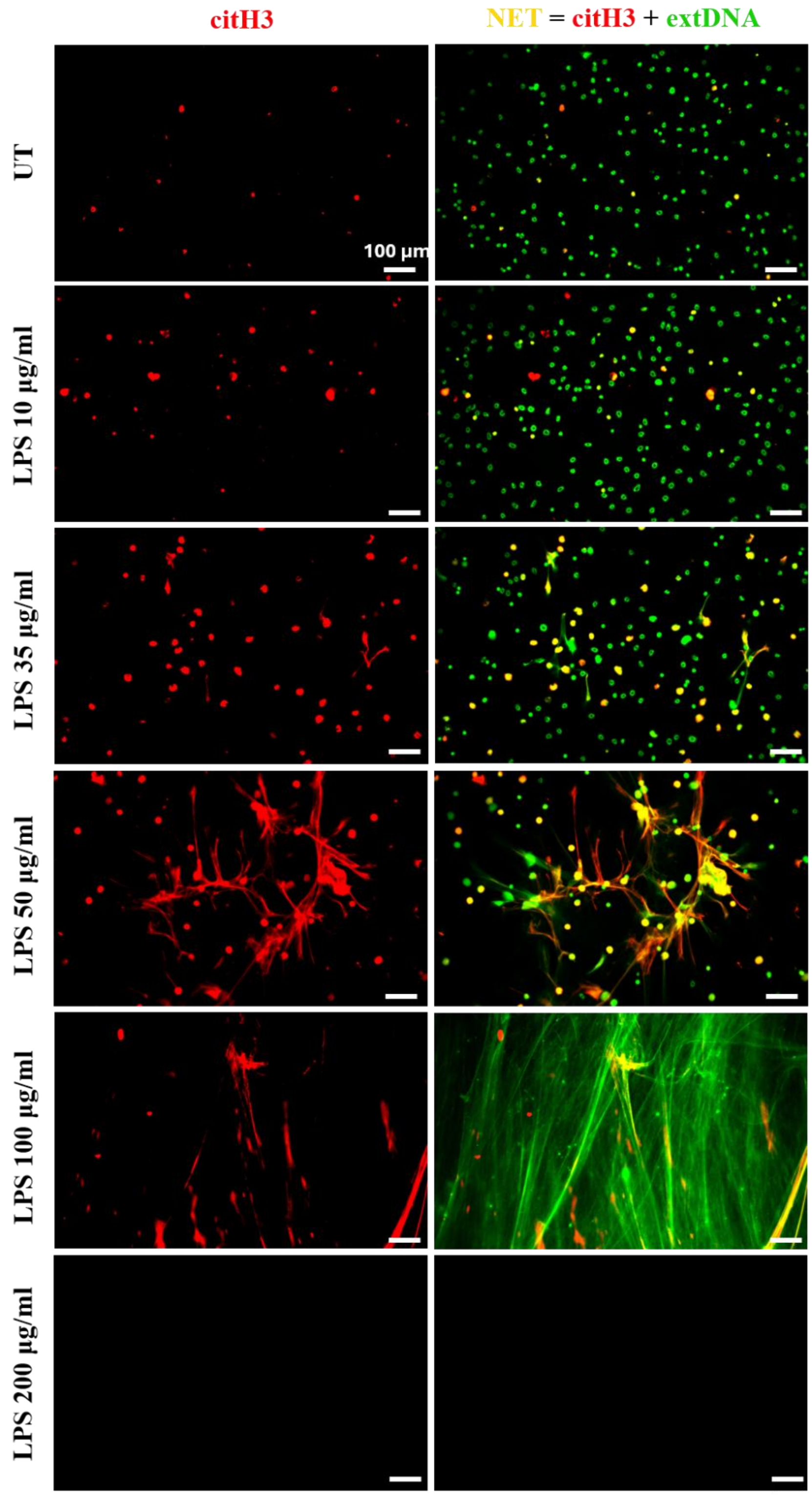

Supplementary Figure 1. Representative images showing NET induction by various concentrations of lipopolysaccharide (LPS) by murine neutrophils after 6 hour stimulation. UT- untreated/not stimulated with LPS. The following LPS concentrations were used: 10, 35, 50, 100 or 200 µg/ml. Green fluorescence marks extracellular DNA (extDNA), while red indicates citrullinated histone H3 (citH3). Merged channels were used to visualize NETs (NET = extDNA + citH3). Scale bar = 100 µm.

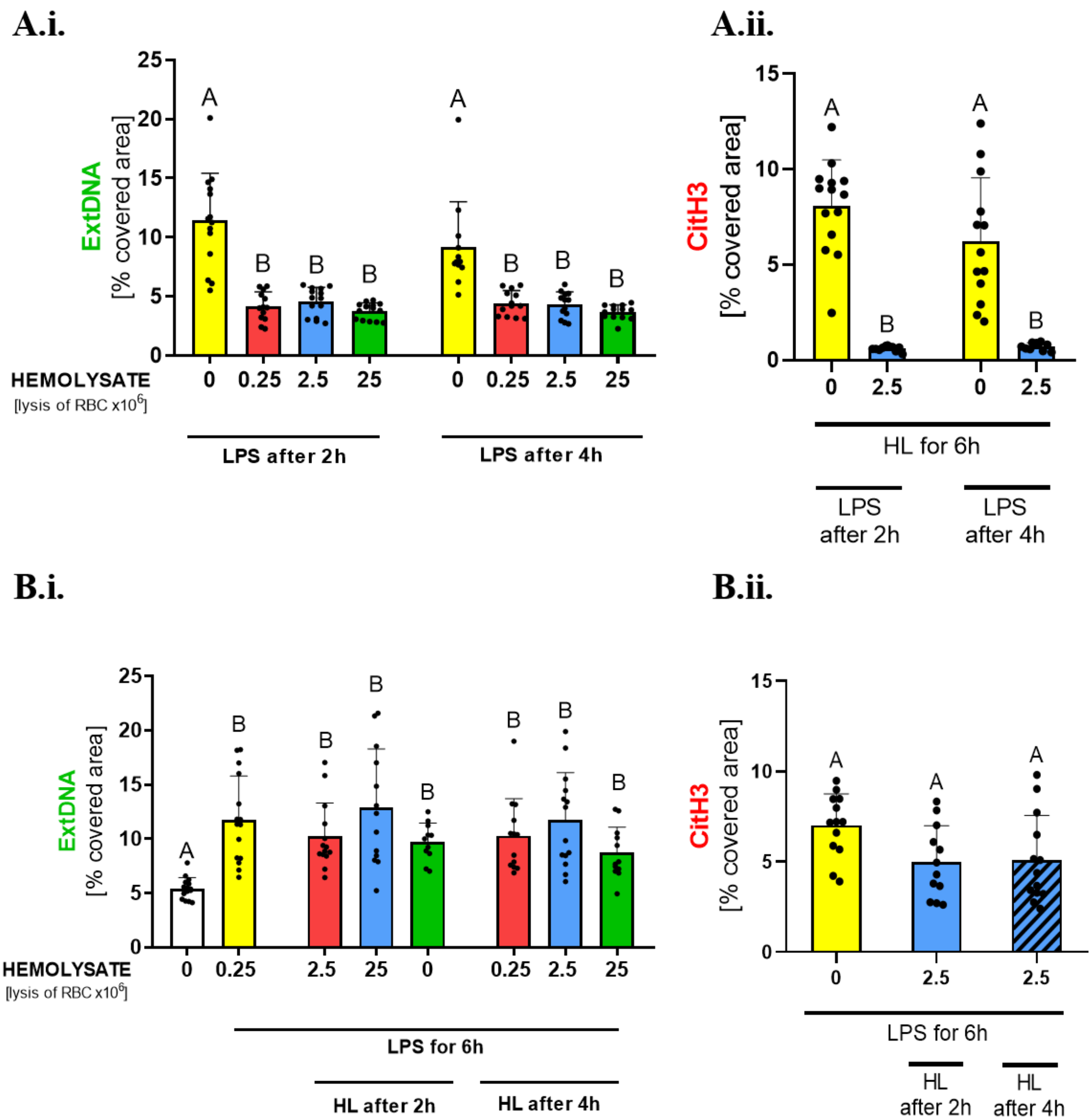

Supplementary Figure 2. Assessment of additional time intervals for adding hemolysate before/after LPS. Cells were incubated with hemolysate (HL) obtained from the lysis of 0.25×10⁶, 2.5×10⁶, or 25×10⁶ red blood cells (RBC), with or without lipopolysaccharide (LPS; 50 µg/ml), or left untreated (0) for 6h. NET formation was measured by the area of FOV covered with extracellular DNA (extDNA) or citrullinated histone H3 (citH3), a marker protein for NETs. Neutrophils were incubated with the indicated concentrations of HL for 6h, with LPS added after 2h or 4h, or with LPS for 6h and HL added after for 2h or 4h. Graphs in the left panel (A.i., B.i.) represent analysis of extDNA, and in the right panel (A.ii.- B.ii.) of citH3. Results are expressed as mean values with SD ($n \geq 3$), where n refers to the number of animals used to collect neutrophils. Within each experiment (cells from one mouse donor), multiple (4-5 on average) fields of view (FOVs) were imaged and analyzed; all raw data (for all FOVs) are presented as dots against the bars (mean value). Differences among groups (A.i. and B.i.) were assessed by Kruskal–Wallis test followed by Dunn's multiple-comparisons applied for post hoc comparisons or (A.ii. and B.ii.) by one-way ANOVA, with Bonferroni correction applied for post hoc comparisons. Statistical significance is indicated by different letters ($p < 0.05$).

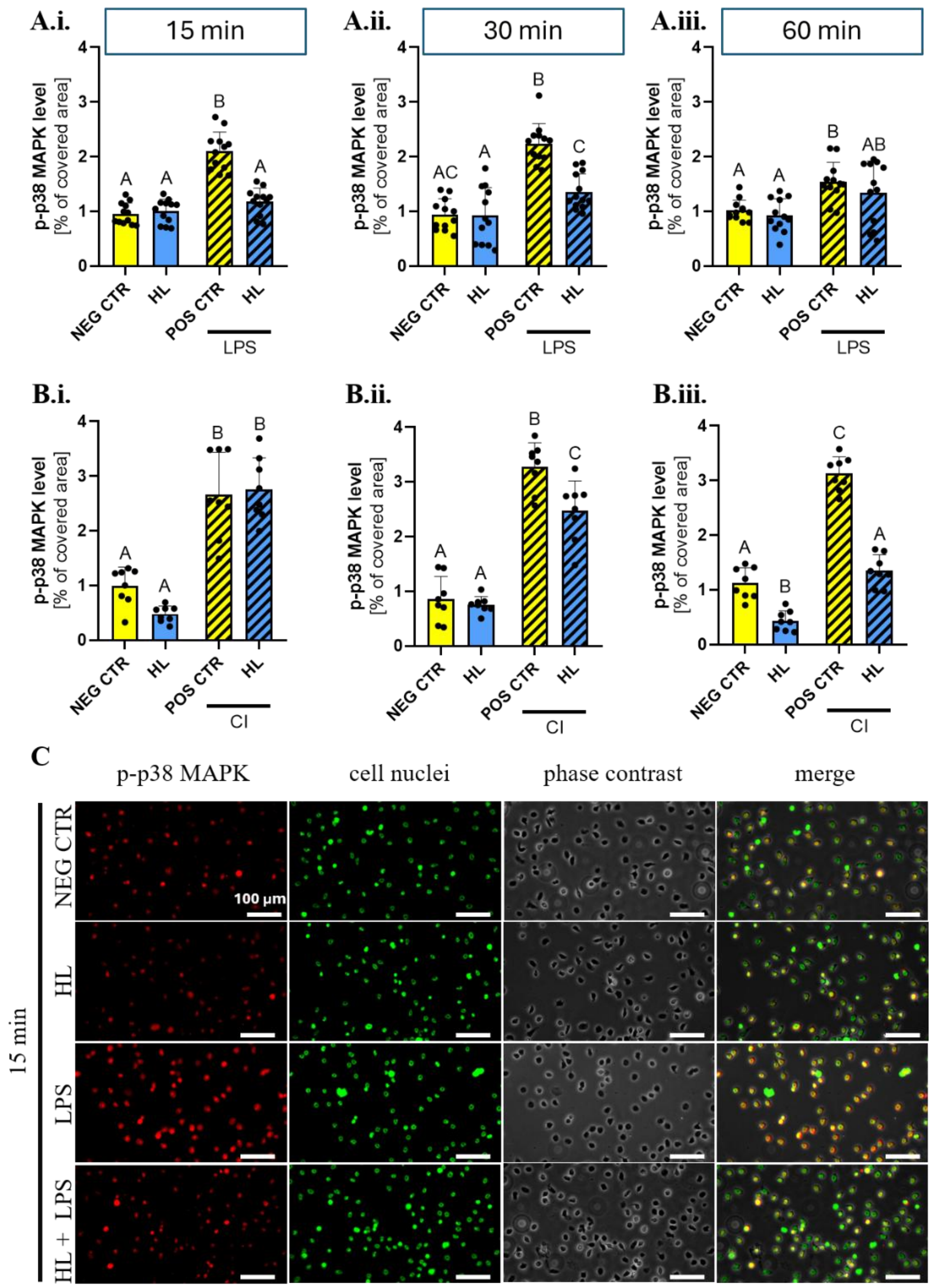

Supplementary Figure 3. Assessment of p38 MAPK phosphorylation after hemolysate and/or LPS in three times intervals. Cells were incubated with hemolysate (HL) obtained from the lysis of 2.5×10^6 red blood cells (RBC), with or without lipopolysaccharide (LPS; 50 µg/ml; POS CTR), calcium ionophore (CI, 10 µM, POS CTR), or left untreated (NEG CTR). Phosphorylation of p38 MAPK (p-p38 MAPK) was measured by the area of FOV covered with this molecule. Neutrophils were incubated with the indicated concentration of HL with LPS for (A.i. – B.i.) 15 min, (A.ii. – B.ii.) 30 min or (A.iii. – B.iii.) 60 min with/without LPS. (C) Representative images of neutrophils treated with LPS and/or HL for 15 min, where red shows level of p-p38 MPAK molecule, green shows extDNA and cells were also visualized by phase contrast. Merge panel are overlay of all channels. Scale bar = 100 µm. Results are expressed as mean values with SD (n = 3 for A; n=2 for B), where n refers to the number of animals used to collect neutrophils. Within each experiment (cells from one mouse donor), multiple (4-5 on average) fields of view (FOVs) were imaged and analyzed; all raw data (for all FOVs) are presented as dots against the bars (mean value). Differences among groups were assessed by one-way ANOVA, with Bonferroni correction applied for post hoc comparisons. Statistical significance is indicated by different letters ($p < 0.05$).

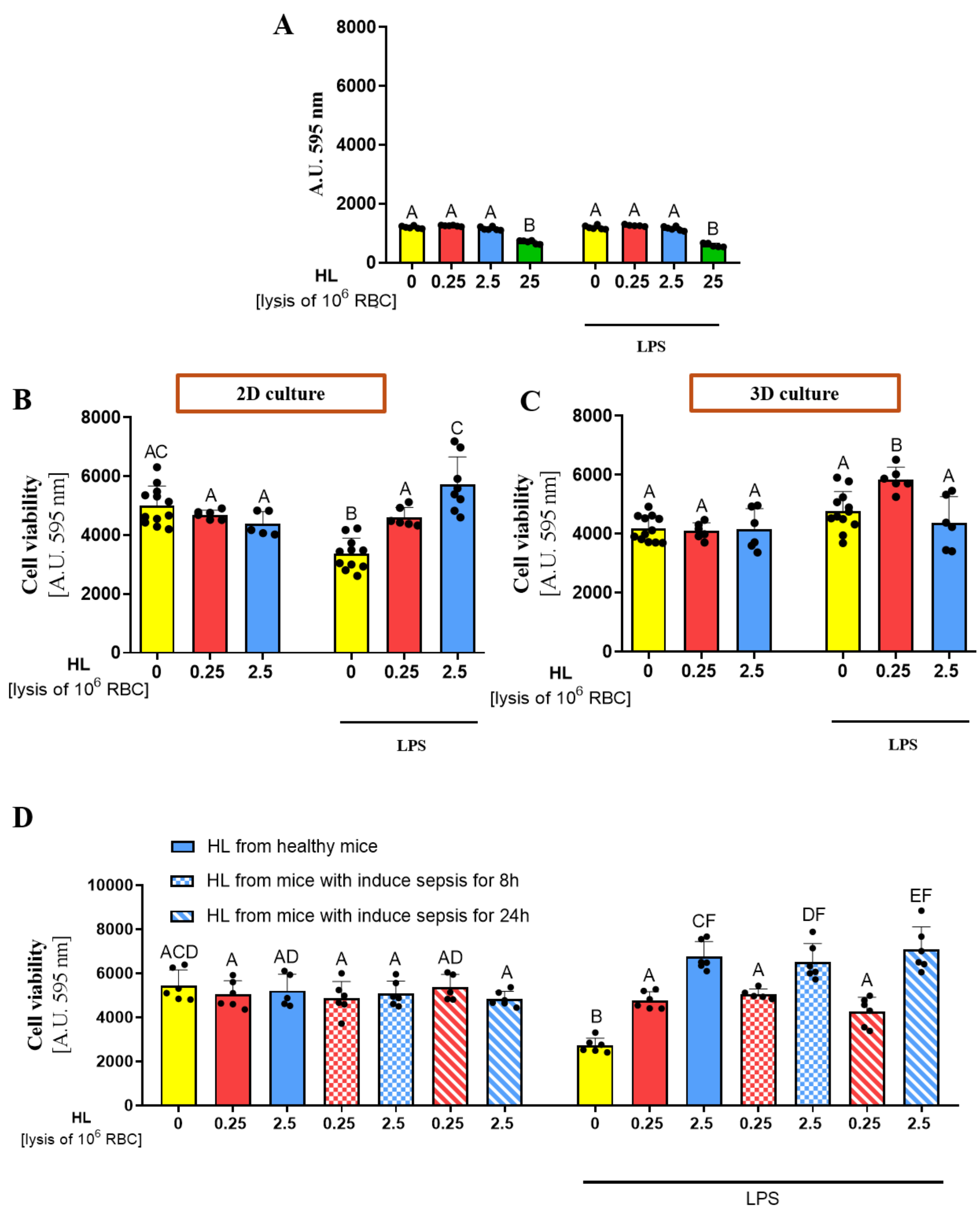

Supplementary Figure 4. The effect of hemolysate on the viability of neutrophils. Viability was measured indirectly by assessing the ability of cells to convert resazurin to resazurin (PrestoBlue assay). (A) Hemolysate was tested alone to rule out any concentration-dependent effects on measurement efficiency. Hemolysate was obtained from 0.25×10^6, 2.5×10^6, or 25×10^6 erythrocytes. Cells were additionally stimulated with lipopolysaccharide (LPS, 35 µg/ml) or left untreated (0) for 6h. (B) Assessment of cell viability after treatment with HL from 0.25×10^6 or 2.5×10^6 erythrocytes and LPS in a 2D system, and (C) data obtained on a 3D matrix. (D) Assessment of cell viability after treatment with HL (from lysis of 0.25×10^6 or 2.5×10^6 erythrocytes) and stimulated with LPS (50 µg/ml) from intact mice and after induction of endotoxemia for 8 or 24 hours. Results are expressed as mean values with SD ($n \geq 3$), where n refers to the number of animals used to collect neutrophils. Differences among groups were assessed by (A-C) one-way ANOVA with Bonferroni correction, (D) or by a non-parametric Kruskal–Wallis test followed by Dunn's multiple-comparisons for post hoc comparisons. Statistical significance is indicated by different letters ($p < 0.05$).

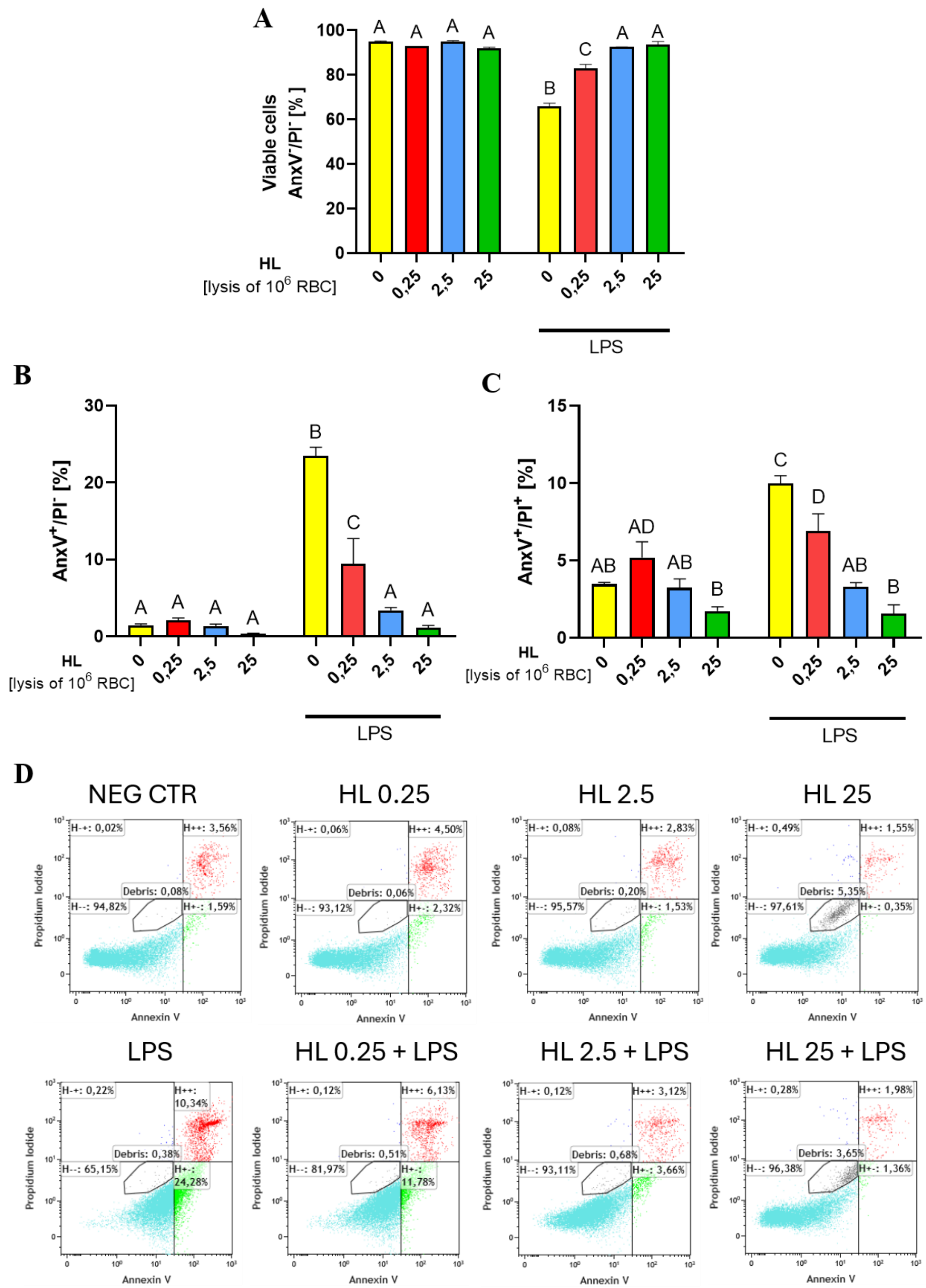

Supplementary Figure 5. The effect of hemolysate on the viability of neutrophils. Cell viability was assessed by flow cytometry using Annexin V (AnxV) and propidium iodide (PI) staining. Cells were incubated (6h) with hemolysate (HL) obtained from the lysis of 0.25×10^6, 2.5×10^6 or 25×10^6 red blood cells (RBC), with or without lipopolysaccharide (LPS; 50 µg/ml) or left untreated (0/NEG CTR) (A) Percentage of viable cells ($AnxV^-/PI^-$), (B) early apoptotic cells (Annexin V^+/PI^-), (C) late apoptotic/secondary necrotic cells (Annexin V^+/PI^+) were assessed. (D) Representative gating strategy used for the analysis. As the highest concentration of hemolysate (obtained from 25×106 RBCs) contained membrane ghosts, an additional flow cytometric gating strategy was implemented to exclude these particles from the analysis. Results are expressed as mean values with SD (n = 2). Differences among groups were assessed by one-way ANOVA with Bonferroni correction. Statistical significance is indicated by different letters ($p < 0.05$).

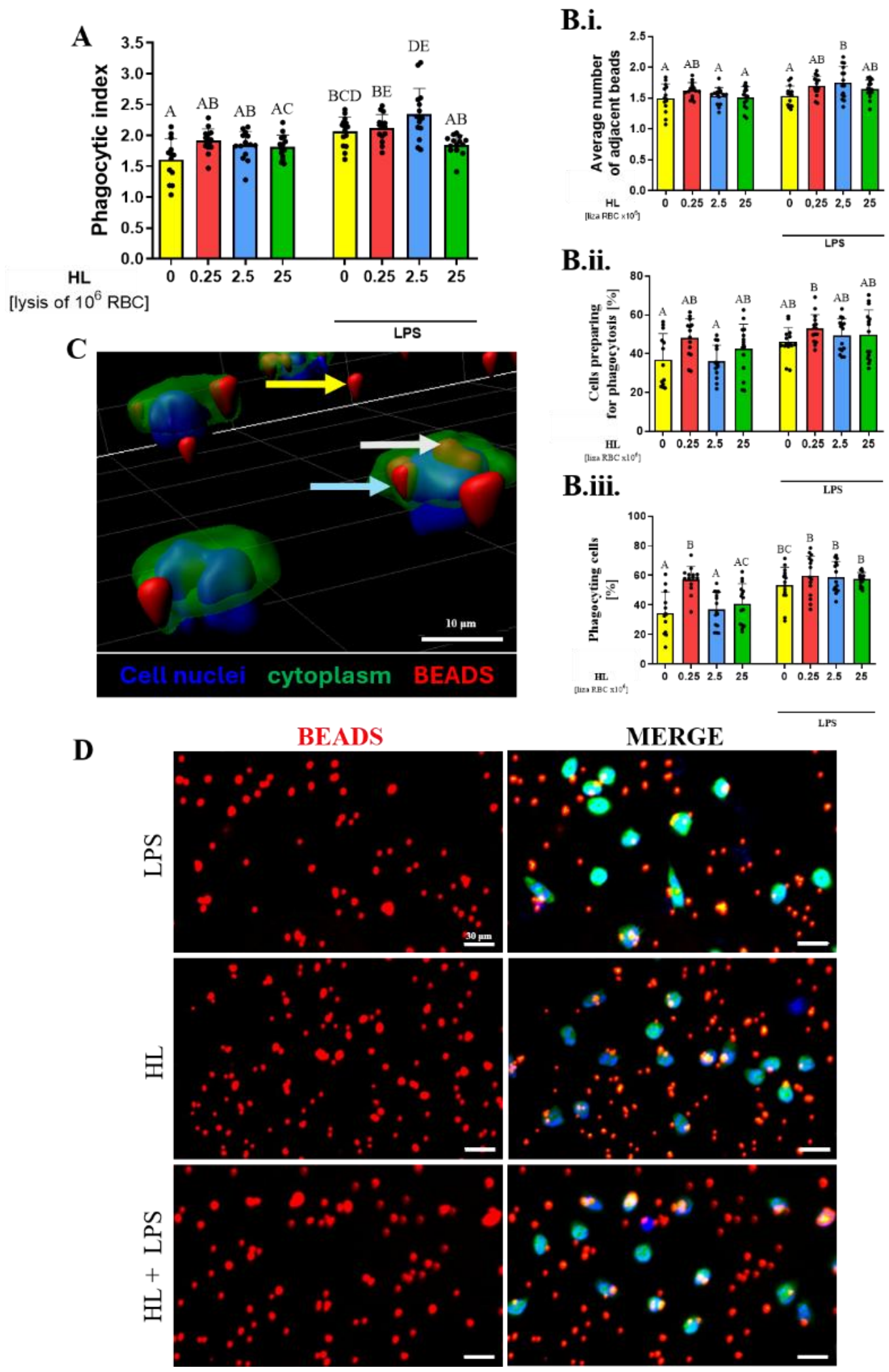

Supplementary Figure 6. The effect of hemolysate on the phagocytic ability of mouse neutrophils and the percentage of cells performing phagocytosis. Hemolysate was obtained from 0.25×10^6, 2.5×10^6, or 25×10^6 erythrocytes. Cells were additionally stimulated with lipopolysaccharide (LPS, 35 g/ml;) or left untreated (0) for 2h. (A) Results of the effect of hemolysate on phagocytic activity, presented using the phagocytic index. (B) Results of the effect of hemolysate on (B.i.) the number of adhering BEADS, (B.ii.) the number of cells preparing for phagocytosis, and (B.iii.) the percentage of phagocytosing cells. (C, D) The red signal was emitted by BEADS latex beads. The green fluorescence came from the cytoplasm of cells labeled with CellTracker Green, while the blue signal came from the cell nucleus labeled with Hoechst dye. The yellow signal came from the mixture of fluorescence emissions from latex beads and cell cytoplasm, indicating their complete phagocytosis by neutrophils. The overlap of the signal (yellow color) indicated phagocytosis of the beads by neutrophils. (C) 3D model showing a spatial image of phagocytosing neutrophils (semi-transparent mode). Arrows: yellow – free BEADS, white – complete phagocytosis, and blue – onset of phagocytosis. Scale bar = 10 µm. (D) Representative images of phagocytosis of BEADS. Scale bar = 30 µm. Results are expressed as mean values with SD ($n \geq 3$), where n refers to the number of animals used to collect neutrophils. Within each experiment (cells from one mouse donor), multiple (4-5 on average) fields of view (FOVs) were imaged and analyzed; all raw data (for all FOVs) are presented as dots against the bars (mean value). Differences among groups were assessed by one-way ANOVA with Bonferroni correction, or (B.i.) by a non-parametric Kruskal–Wallis test followed by Dunn's multiple-comparisons for post hoc comparisons. Statistical significance is indicated by different letters ($p < 0.05$).

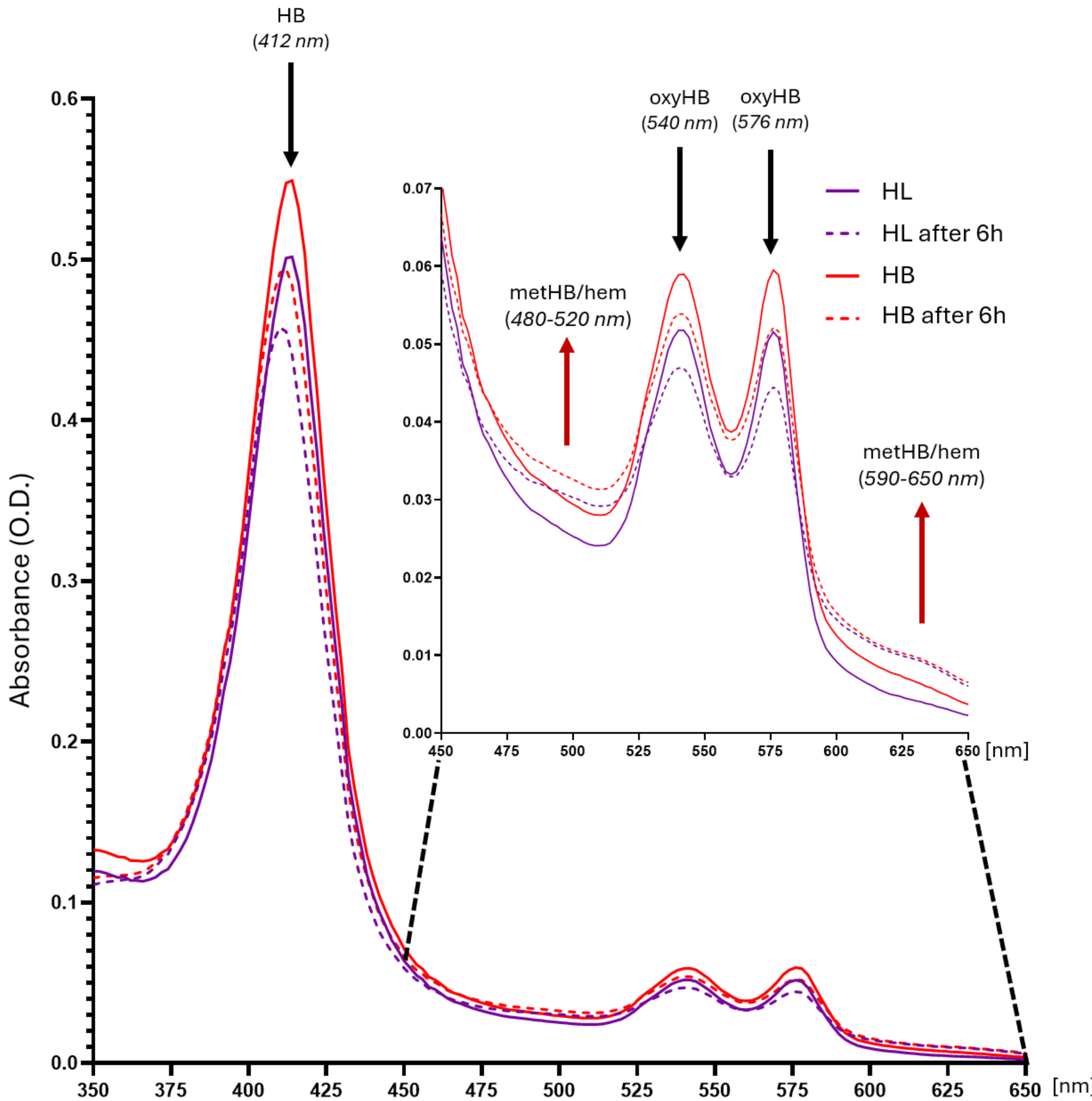

Supplementary Figure 7. Identification of hemolysate and hemoglobin as impermanent reagents and the presence of hemoglobin disintegration products. Hemoglobin (HB, 0.3 mg/mL, red line) and hemolysate (HL, from lysis of 2.5×10^6 RBC; purple line) were measured at time 0 (solid line) and after 6h of incubation (dotted line). The absorbance scan was performed from 350 to 650 nm in 2 nm increments. Black arrows indicate decreases in peaks specific to HB, and red arrows indicate increases in signal from metHB/heme – products of HB disintegration.

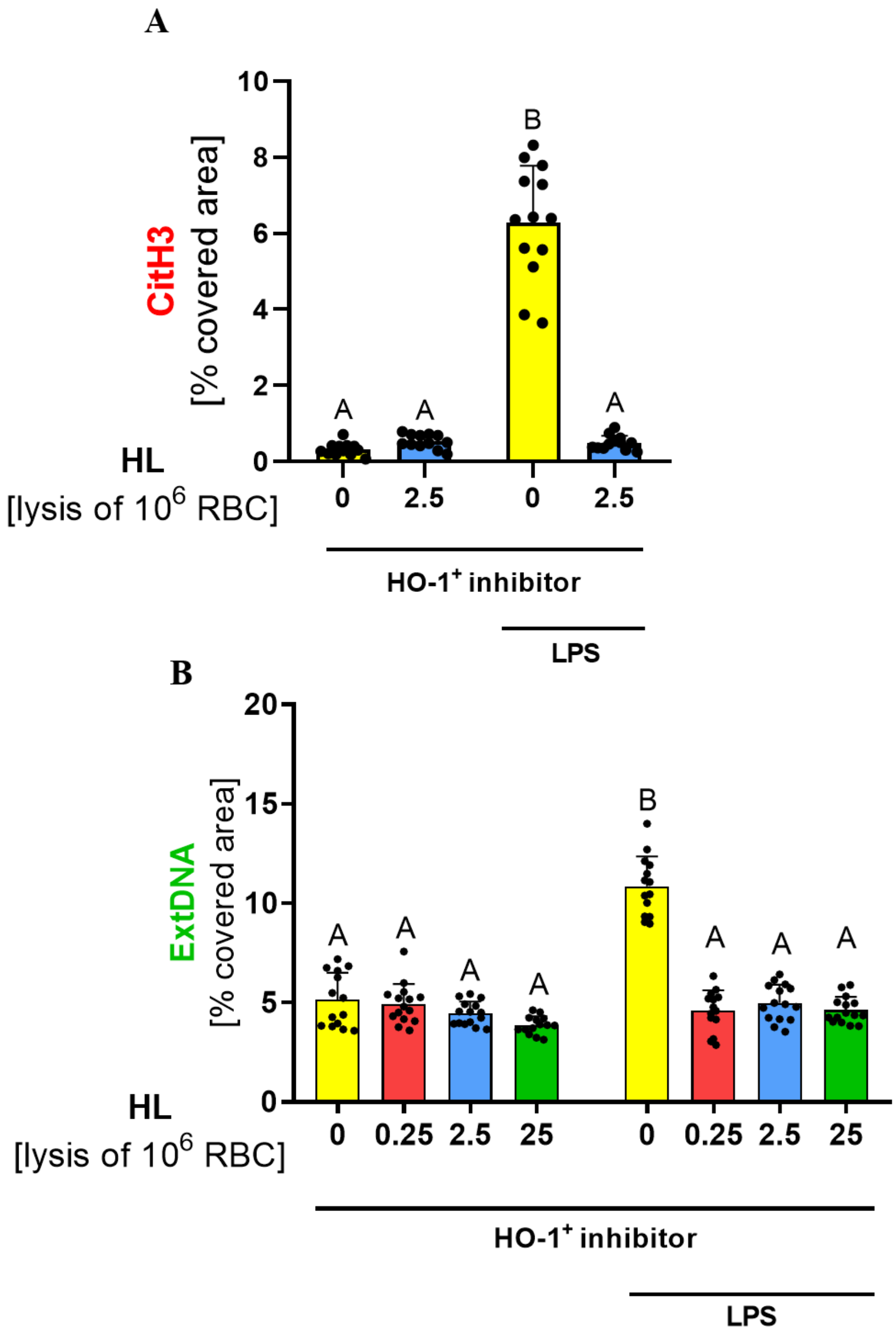

Supplementary Figure 8. Effect of the hemolysate on NET formation in neutrophils with blocked HO-1 signaling. Neutrophils were pretreated with the HO-1 inhibitor (tin protoporphyrin IX dichloride, 20 µM) for 0,5 h and then incubated with hemolysate (HL) obtained from the lysis of 0.25×10^6, 2.5×10^6, or 25×10^6 red blood cells (RBC) with or without lipopolysaccharide (LPS; 50 µg/ml) treatment, or left untreated (0) for 6 h. NET formation was measured as the area of the FOV covered by (A) citrullinated histone (citH3) and (B) extracellular DNA (extDNA). Results are expressed as mean values with SD ($n \geq 3$), where n refers to the number of animals used to collect neutrophils. Within each experiment (cells from one mouse donor), multiple (4-5 on average) fields of view (FOVs) were imaged and analyzed; all raw data (for all FOVs) are presented as dots against the bars (mean value). Differences among groups were assessed by (A) one-way ANOVA with Bonferroni correction for post hoc comparisons or (B) Kruskal–Wallis test followed by Dunn's multiple-comparisons test for post hoc comparisons. Statistical significance is indicated by different letters ($p < 0.05$).

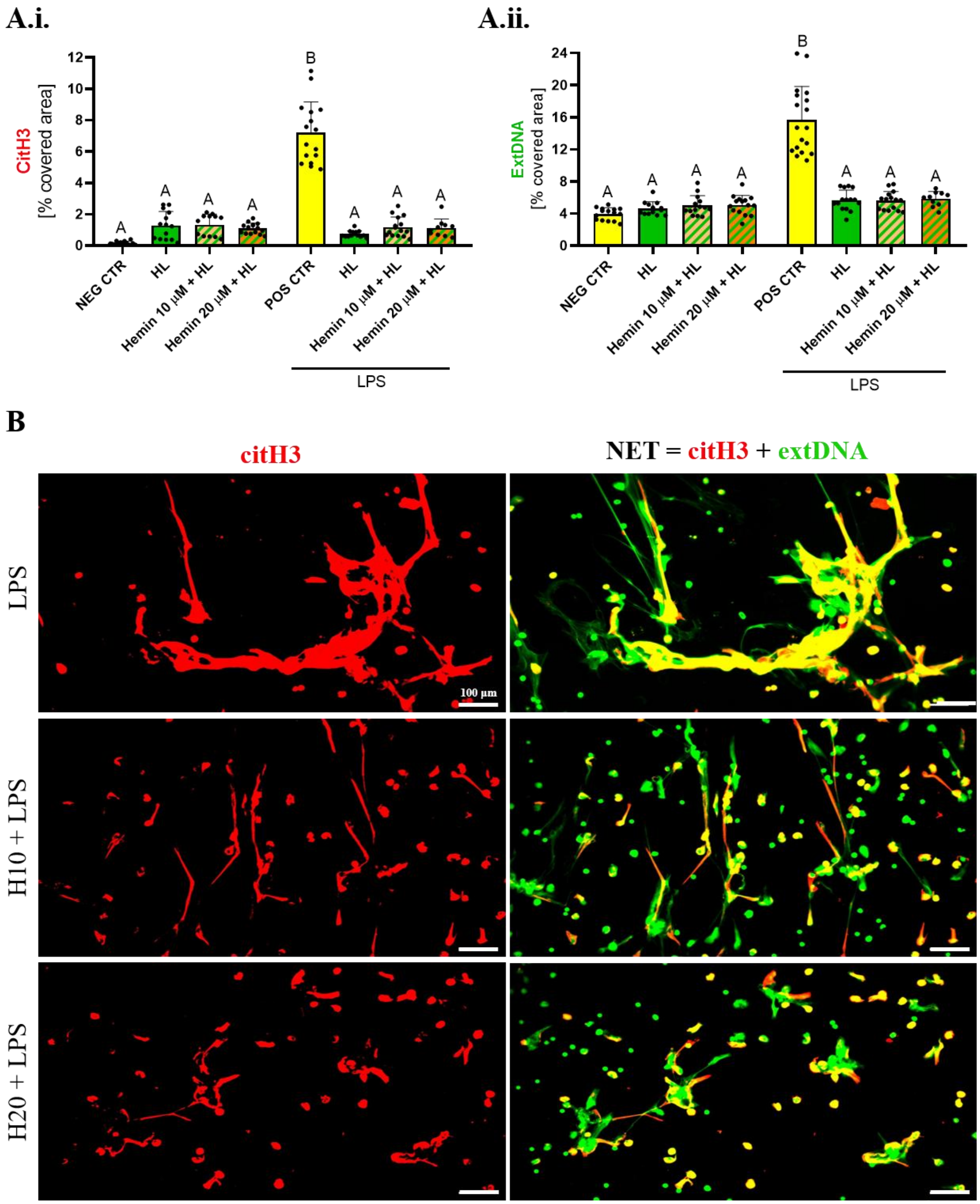

Supplementary Figure 9. Effect of hemin, hemoglobin, or hemolysate on NET formation. (A) Neutrophils were exposed to hemin at two concentrations (10 and 20 µM; H10/H20) alone or with lipopolysaccharide (LPS; 50 µg/ml, POS CTR), with or without HL ($25x10^6$), or left untreated (NEG CTR) for 6 h. In all experimental setups, incubation lasted 6h. NET formation was measured by the area of the FOV covered with extracellular DNA (extDNA) or citrullinated histone H3 (citH3), a marker protein for NETs. Graph (A.i.) represents analysis of citH3, and (A.ii.) of extDNA. (B) Representative images of neutrophils obtained from groups treated with LPS alone or with two tested hemin concentrations. Green fluorescence marks extDNA, while red indicates citH3. Merged channels were used to visualize NETs (NET = extDNA + citH3). Scale bar = 100 µm. Results are expressed as mean values with SD ($n \geq 3$). Within each experiment (cells from one mouse donor), multiple (4-5 on average) fields of view (FOVs) were imaged and analyzed; all raw data (for all FOVs) are presented as dots against the bars (mean value). Differences among groups were assessed by one-way ANOVA with Bonferroni correction for post hoc comparisons. Statistical significance is indicated by different letters ($p < 0.05$).

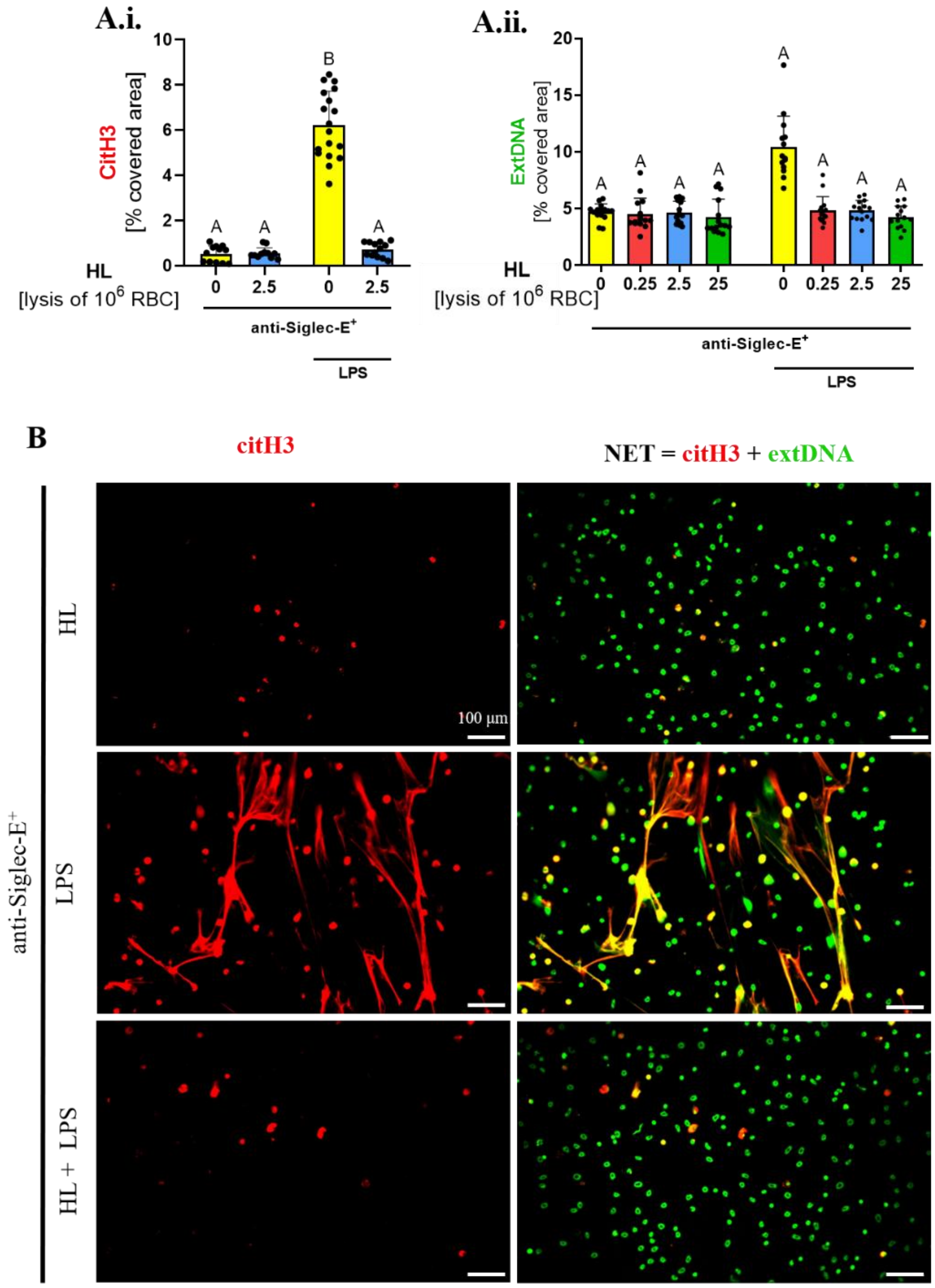

Supplementary Figure 10. Effect of the hemolysate on NET formation in neutrophils with blocked Siglec-E signaling. Neutrophils were pretreated with (A) anti-Siglec-E antibody for 30 min and then incubated with hemolysate (HL) obtained from lysis of 0.25×10^6, 2.5×10^6, or 25×10^6 red blood cells (RBC) with or without lipopolysaccharide (LPS; 50 µg/ml) treatment, or left untreated (0) for 6 h. NET formation was measured as the area of FOV covered by (A.i.) citrullinated histone (citH3) and (A.ii.) extracellular DNA (extDNA). (B) Representative images of neutrophils with blocked Siglec-E signaling, where green indicates extDNA and red indicates citH3 (images from both channels were merged to visualize NETs; NET = extDNA + citH3; HL concentration equals 2.5×10^6 lysed RBC). Scale = 100 µm. Results are expressed as mean values with SD ($n \geq 3$). Within each experiment (cells from one mouse donor), multiple (4-5 on average) fields of view (FOVs) were imaged and analyzed; all raw data (for all FOVs) are presented as dots against the bars (mean value). Differences among groups were assessed by (A.i.) one-way ANOVA with Bonferroni correction for post hoc comparisons or by (A.ii.) Kruskal–Wallis test followed by Dunn's multiple-comparisons for post hoc comparisons. Statistical significance is indicated by different letters ($p < 0.05$). Statistical significance is indicated by different letters ($p < 0.05$).

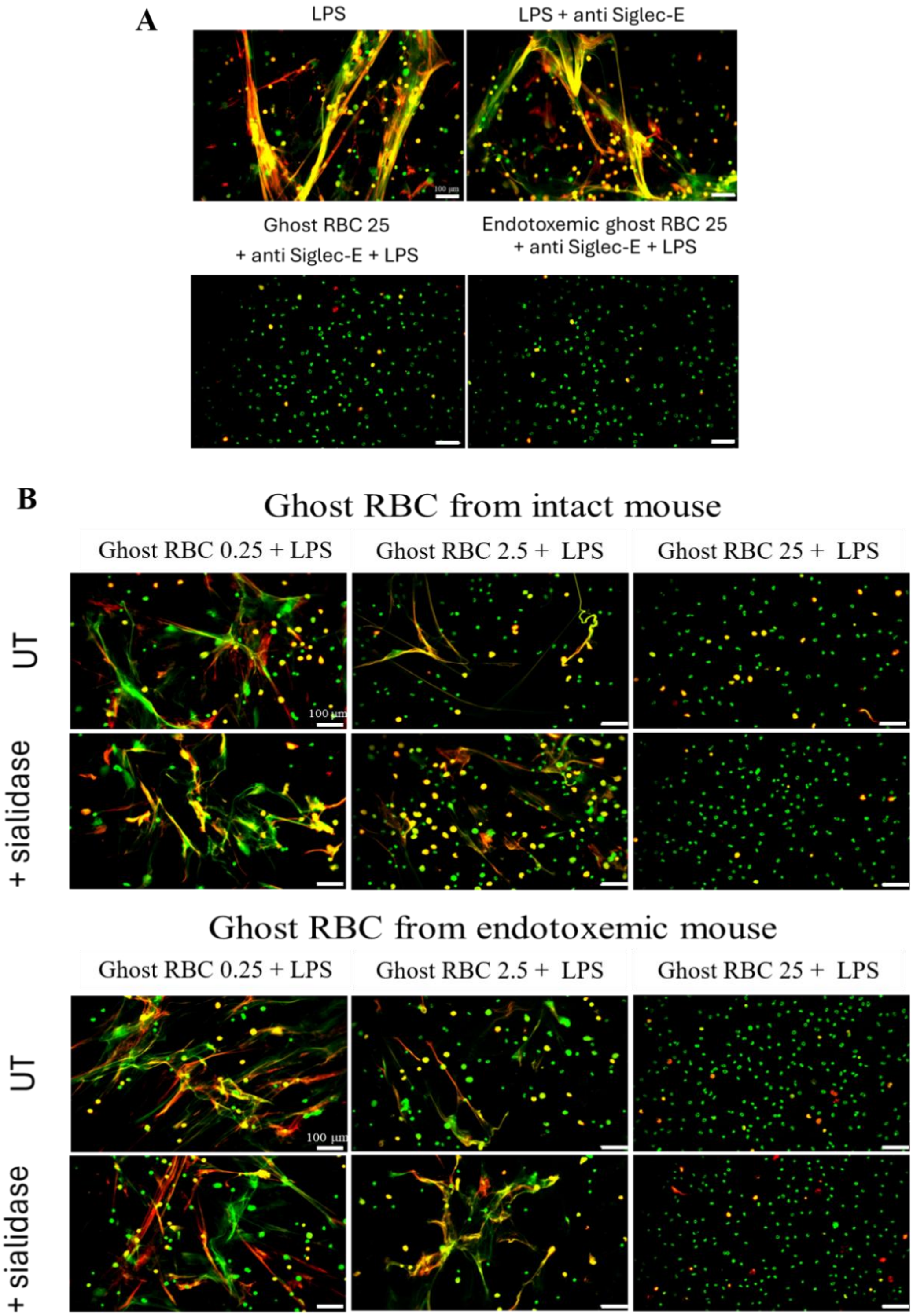

Supplementary Figure 11. Representative images of NET formation after PMN co-incubation with ghost RBCs and anti-SiglecE antibody or with sialic acid-depleted ghosts. (A) Neutrophils were exposed to RBC ghosts (from lysis of 25×10^6 cells) alone or with lipopolysaccharide (LPS; 50 µg/ml). PMN were incubated for 0,5h with anti-Siglec-E antibody before co-incubation with ghosts. (B) Neutrophils were exposed to RBC ghosts (from lysis of 0.25×10^6, 2.5×10^6, or 25×10^6 cells) alone or with LPS, or left untreated (UT). Some ghosts were also incubated with sialidase for 1h before co-incubation with neutrophils. Green fluorescence marks extDNA, while red indicates citH3. Merged channels were used to visualize NETs (NET = extDNA + CitH3). Scale bar = 100 µm.

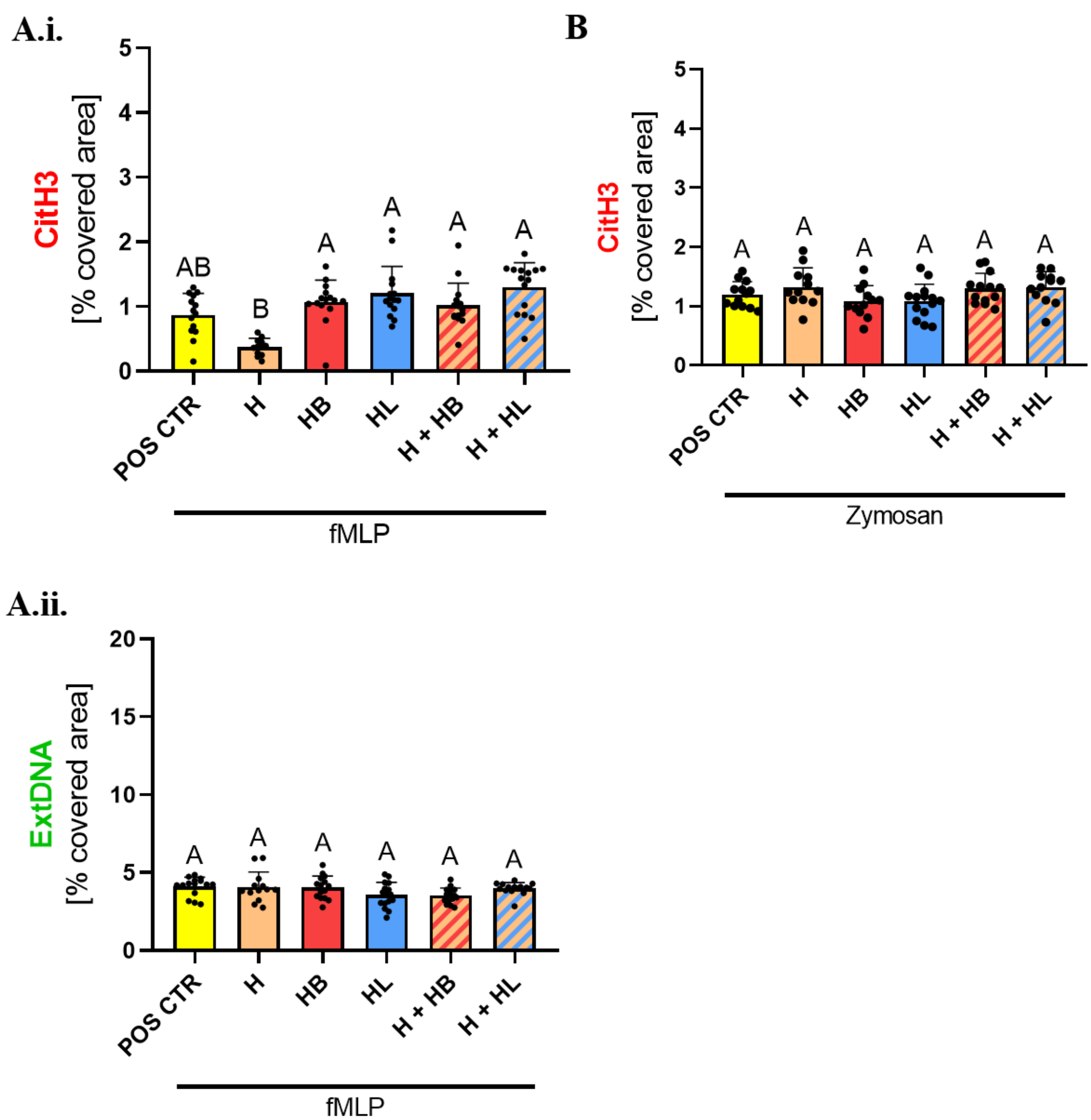

Supplementary Figure 12. Quantification of NET formation after exposure to hemolysate and/or its components fMLP and zymosan. All experiments in this configuration were performed in RPMI 1640 medium. Cells were exposed to hemoglobin (HB; 0.3 mg/ml), hemin (H; 10 µM), or hemolysate (HL from lysis of 2.5×10^6 red blood cells), either alone or in combination (H+HL/H+HB). In addition, neutrophils were treated with fMLP (1 µM) or Zymosan (100 µg/ml). (A) Quantification of NETs formed by neutrophils from WT mice treated with fMLP. NETs were stained, and FOVs were quantified for the area of (A.i.) citrullinated histone (citH3) and (A.ii.) extracellular DNA (extDNA). (B) Neutrophils were treated with H, HL, or both, and with zymosan NETs were stained, and FOVs were quantified only by the area of citH3 due to nonspecific signal from zymosan particles in green fluorescence channel. Results are expressed as mean values with SD ($n \geq 3$), where n refers to the number of animals used to collect neutrophils. Within each experiment (cells from one mouse donor), multiple (4-5 on average) fields of view (FOVs) were imaged and analyzed; all raw data (for all FOVs) are presented as dots against the bars (mean value). Differences among groups were assessed by one-way ANOVA with Bonferroni correction for post hoc comparisons. Statistical significance is indicated by different letters ($p < 0.05$).

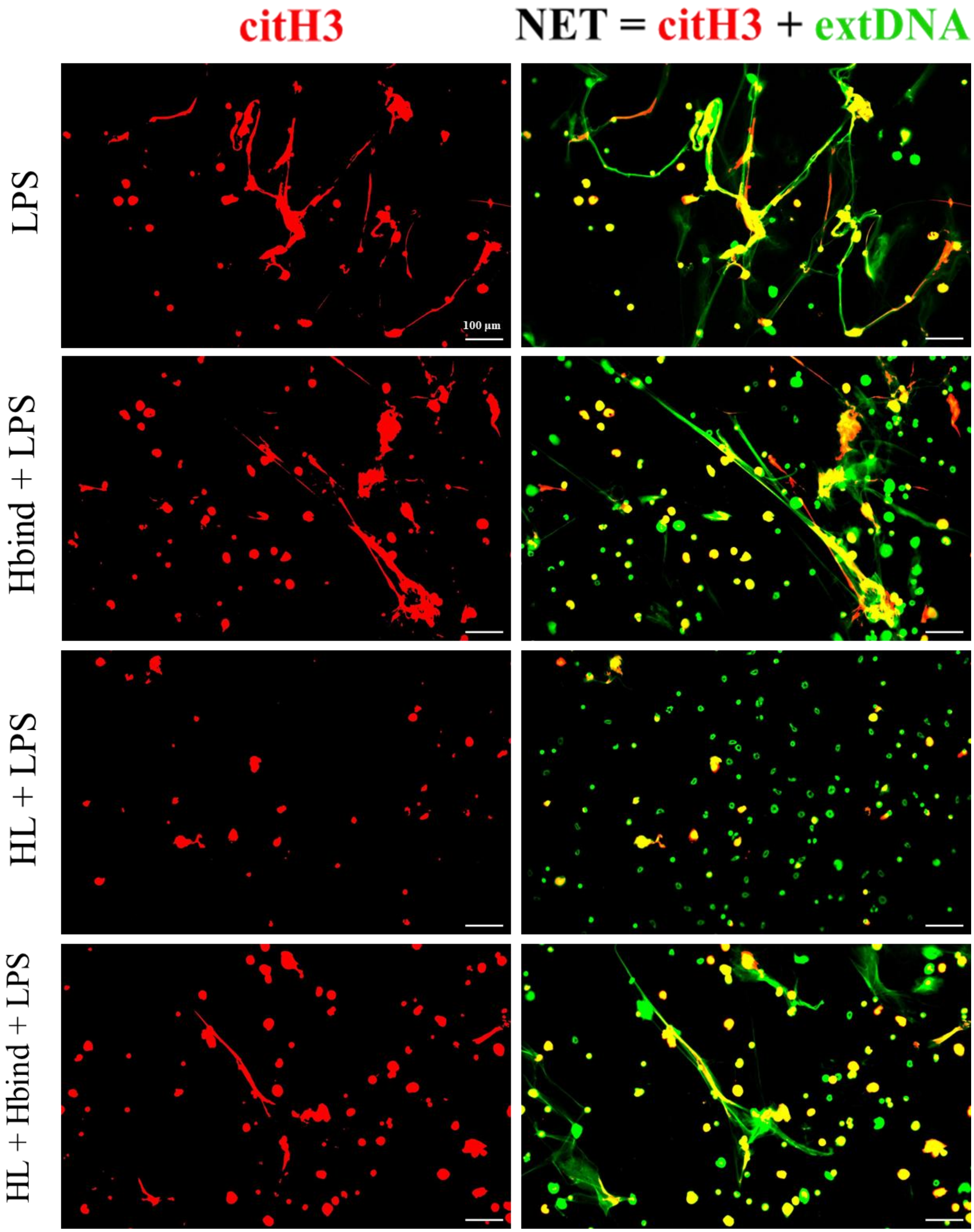

Supplementary Figure 13. Evaluation of the role of hemoglobin in NET suppression. Hemoglobin (HB) was depleted from HL (2.5×10^6 cells) using HemogloBind (HBind) and ghosts with centrifugation, leaving other components intact. Cells were incubated with HL (2.5×10^6 cells), HBind (2.5×10^6 cells), with or without lipopolysaccharide (LPS) at 50 µg/ml. Green fluorescence marks extDNA, while red indicates citH3. Merged channels were used to visualize NETs (NET = extDNA + CitH3). Scale bar = 100 µm.

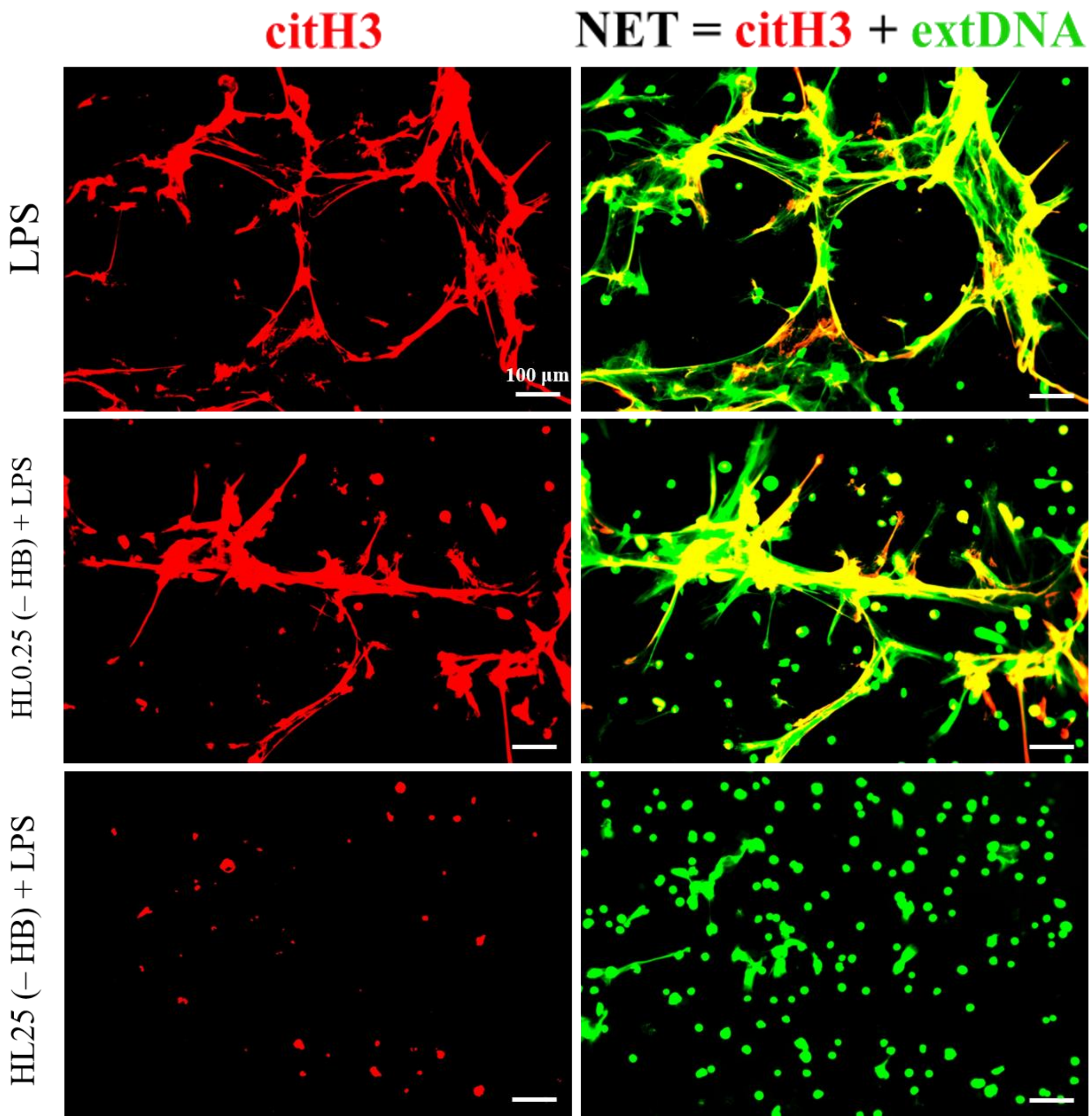

Supplementary Figure 14. The effect of non-specific removal of hemoglobin from HL on NET formation. Hemoglobin (HB) was depleted from HL (0.25×10^6 or 25×10^6 cells; HL - HB) using zinc chloride. Cells were incubated with HL after HB precipitation, with or without lipopolysaccharide (LPS) at 50 µg/ml. Green fluorescence marks extDNA, while red indicates citH3. Merged channels were used to visualize NETs (NET = extDNA + CitH3). Scale bar = 100 µm.

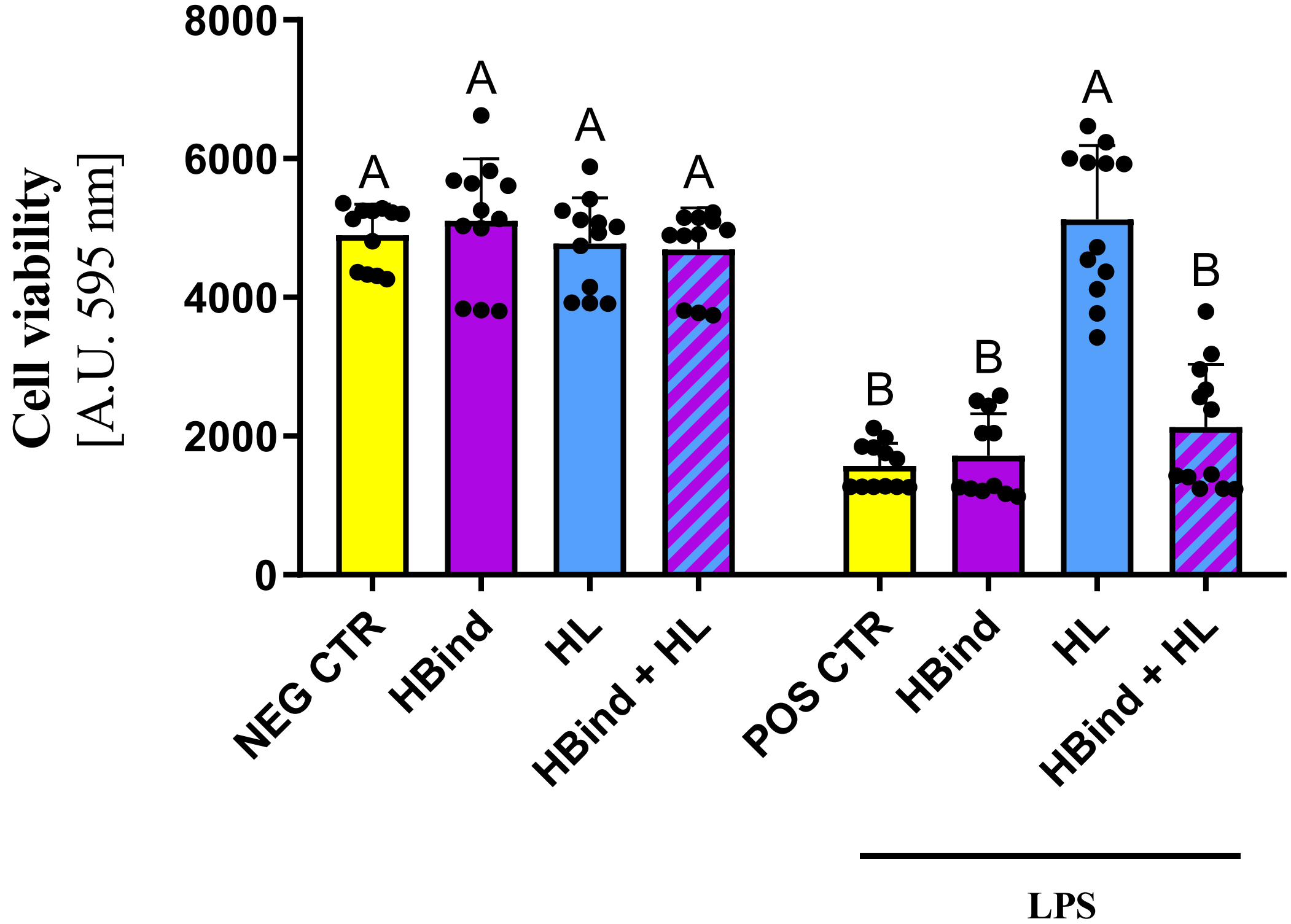

Supplementary Figure 15. The effect of hemoglobin and the removal of hemoglobin and ghosts from HL on the viability of neutrophils. Viability was measured indirectly by assessing the ability of cells to convert resazurin to resazurin (PrestoBlue assay). Cell viability after removal of hemoglobin and ghosts (2.5×10^{6}) from HL using the HemogloBind (HBind) reagent and high-speed centrifugation, with lipopolysaccharide (LPS) at 50 µg/ml (POS CTR). Results are expressed as mean values with SD ($n \geq 3$). Differences among groups were assessed by one-way ANOVA with Bonferroni correction. Statistical significance is indicated by different letters ($p < 0.05$).

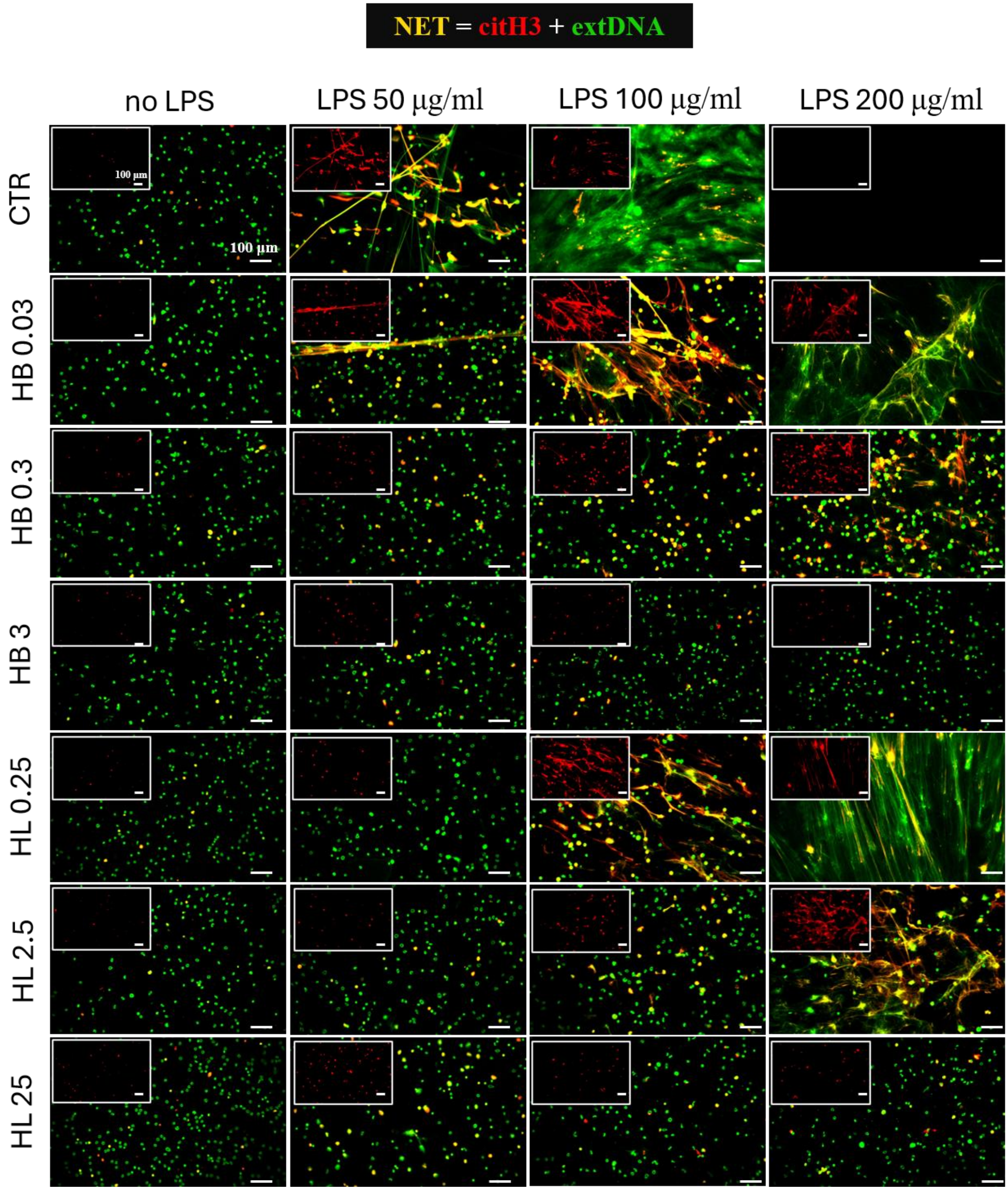

Supplementary Figure 16. Representative images showing the capacity of high concentrations of lipopolysaccharide (LPS) to induce NET formation by murine neutrophils after 6h hour stimulation in the presence of hemolysate (HL) or hemoglobin (HB). Green fluorescence marks extDNA, while red indicates citH3. Merged channels were used to visualize NETs (NET = extDNA + citH3). Inserts show signal for citH3 only. CTR – controls without either HL or HB. The following concentrations were used: LPS (50, 100 or 200 μg/ml), HL (0.03; 0.3 or 3 mg/ml) and HB (0.25×10^6; 2.5×10^6 or 25×10^6). Scale bar = 100 μm.

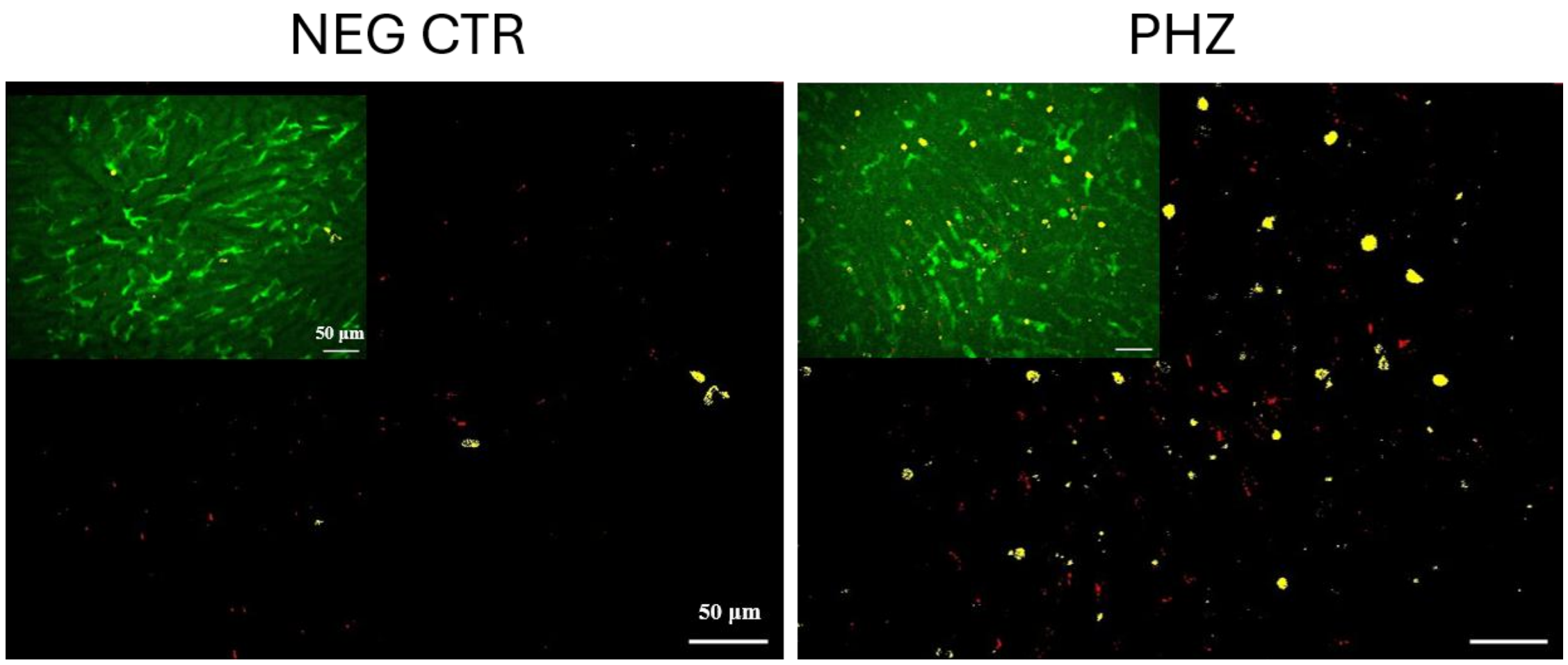

Supplementary Figure 17. Representative images of histone H2A.X area and neutrophil number in control groups for observation of endotoxemia induced in hemolytic mice. C57BL/6J mice were treated with phenylohydrazine (PHZ) to induce hemolysis. PHZ was injected intraperitoneally twice, at time 0 (40 mg/kg body weight; b.w.) and after 12 h (60 mg/kg b.w.). The images show the livers of mice treated with PHZ alone, where false yellow color indicates Ly6G+ neutrophils (originally BV421) and violet fluorescence indicates H2A.X histones (AF647) – a marker of NETs – or of mice not treated at all (NEG CTR). In the upper-left corner of the images, the whole liver is shown, with additional dark green signal from hepatocytes and light green from anti-F4/80-stained Kupffer cells. Scale bar = 50 µm.
